# Chronic activation of JNK JAK/STAT and oxidative stress signalling causes the loser cell status

**DOI:** 10.1038/s41467-017-00145-y

**Published:** 2017-07-26

**Authors:** Iwo Kucinski, Michael Dinan, Golnar Kolahgar, Eugenia Piddini

**Affiliations:** 1 The Wellcome Trust/Cancer Research UK Gurdon Institute and Zoology Department, University of Cambridge, Tennis Court Road, Cambridge, CB2 1QN UK; 2 School of Cellular and Molecular Medicine, University of Bristol, Biomedical Sciences Building, University Walk, Bristol, BS8 1TD UK; 3 Wellcome Trust and MRC Cambridge Stem Cell Institute, Department of Haematology and Cambridge Institute of Medical Research, University of Cambridge, Hills Road, Cambridge, CB2 0XY UK

## Abstract

Cell competition is a form of cell interaction that causes the elimination of less fit cells, or losers, by wild-type (WT) cells, influencing overall tissue health. Several mutations can cause cells to become losers; however, it is not known how. Here we show that *Drosophila* wing disc cells carrying functionally unrelated loser mutations (*Minute* and *mahjong*) display the common activation of multiple stress signalling pathways before cell competition and find that these pathways collectively account for the loser status. We find that JNK signalling inhibits the growth of losers, while JAK/STAT signalling promotes competition-induced winner cell proliferation. Furthermore, we show that losers display oxidative stress response activation and, strikingly, that activation of this pathway alone, by Nrf2 overexpression, is sufficient to prime cells for their elimination by WT neighbours. Since oxidative stress and Nrf2 are linked to several diseases, cell competition may occur in a number of pathological conditions.

## Abstract (web summary)

Cell competition causes the removal of less fit cells (‘losers’) but why some gene mutations turn cells into losers is unclear. Here, the authors show that *Drosophila* wing disc cells carrying some loser mutations activate Nrf2 and JNK signalling, which contribute to the loser status.

## Introduction

The health of tissues and the quality of its cellular constituents is actively maintained by a range of cell–cell interactions, known as cell competition. Through cell competition cells sense fitness level heterogeneities across cell populations and this results in the elimination of the less fit cells (or losers) when they are in the presence of fitter cells (or winners), in a process akin to natural selection.

Cell competition was originally described in *Drosophila* wing imaginal discs^1^; however, it is now clear that it is a universal process, which also occurs in other *Drosophila* tissues^2, 3^ including adult tissues^4–6^, in the mouse embryo^7–9^, liver^10^ and heart^11^ and in mammalian cell culture^8, 9, 12, 13^. Further studies have also shown the existence of this process in several stem cell compartments^14–17^, although it is likely that in this case it happens via a different mechanism(s).

The discovery of cell competition emerged from *Drosophila* studies of heterozygous mutations in ribosomal genes known as *Minute* mutations^18^. While *Minute* heterozygous cells and animals are viable, in mosaic tissues *Minute* heterozygous cells behave as losers and are killed when confronted with wild-type (WT) cells, allowing the healthy WT population to expand efficiently^1, 19^. In addition to *Minute*, many other mutations have been shown to induce a loser status against WT cells, such as mutations in the oncogene *myc* ^20^, in the polarity genes *scribble* ^2^, *dlg* ^21^ and *lgl* ^22^, in the polarity-associated/Cul4-DDB1 complex component *mahjong* (*mahj*)^12^ and in genes associated with many signalling pathways such as BMP^8, 23^, JAK/STAT^24^, Wingless^6, 25^ and EGF^26^.

Many of the mutations associated with the loser status typically compromise cell growth (as is the case for *Minute* or *myc* mutations), architecture (like mutations in polarity genes) or cell-fate specification (e.g., mutations in BMP, JAK/STAT and Wingless components) and cells harbouring some of these defects show signs of stress, such as activation of the JNK pathway^27^. It is therefore likely that cell competition prevents the accumulation of stressed or mis-specified cells, which could compromise tissue robustness/health or contribute to developmental defects.

Despite these significant implications, the molecular mechanisms underlying cell competition are still not well understood. However, it is clear that three factors contribute to this process and to the selective colonisation of tissues by winner cells. First, loser cells commonly exhibit slower proliferation rates than their winner counterparts and this passively contributes to expansion of the winner cell population^1, 19^. Secondly, it has been reported that during cell competition winner cells further increase their proliferation rates over their already faster baseline^5, 28–31^. It is unclear how that is elicited; however, it has been proposed to be a consequence of winner/loser recognition or simply a compensatory mechanism triggered by loser cell death^28–34^. The third and most striking aspect of cell competition is that loser cells are eliminated in the presence of their fitter neighbours^1, 19^, mostly (but not exclusively) via induction of apoptosis^5, 23, 31, 35^. Collectively, the combination of these three processes, results in cell competition and in the effective colonisation of tissues by winners at the expense of losers. Several molecules, such as Flower^32^, Azot^36^, the Toll/IMD pathway^37^, and the Sas/PTP10D ligand-receptor complex^38^ have been implicated in triggering the apoptosis of losers. However, it is entirely unknown what pre-existing conditions and differences between winners and cells with reduced competitive ability are responsible for initiating the process.

In this study, we sought to identify pre-existing conditions in prospective loser cells that could contribute to their loser status and to cell competition. Using *Drosophila* imaginal wing discs, we took a transcriptomics approach to identify genes and pathways that might be differentially active in cells with reduced competitive ability in their naive state, i.e., before exposure to prospective winner cells. Our data show that cells with mutations in functionally unrelated loser genes share a common molecular signature. Analysis of this signature shows that prospective loser cells chronically activate several stress response pathways, including the JNK and JAK/STAT pathways and many genes involved in the oxidative stress response, which are likely targets of the transcription factor Nrf2. Importantly, we find that these pathways play key roles in cell competition and act as distinct modules to induce the three main features of the competition process, i.e. slow proliferation of losers, over proliferation of winners and loser cell elimination, respectively. Importantly, we find that Nrf2 activity plays a dual role: it promotes autonomous cell survival of *Minute* cells. However, and strikingly, it is also sufficient to prime cells as losers when they are competing against WT neighbours. These findings provide the first insight into the pathways that earmark cells as losers and into the early steps of cell competition.

## Results

### Prospective loser cells share a common molecular signature

To identify genes involved in cell competition, we looked for differences at the gene expression level between WT wing discs (Supplementary Fig. 1a, b) and wing discs mutant for several loser-linked gene mutations (Supplementary Fig. 1c–h). In particular, to identify factors that are responsible for initiating cell competition, we looked for gene expression differences between prospective winner and loser cells in the absence of cell competition. First, we compared the transcriptome of WT cells to that of cells carrying two distinct alleles of the ribosomal gene *RpS3* (denoted as *RpS3* and RpS3*). Both alleles are mutations of the *Minute* gene *M(3)95A* and confer a loser status^18, 39^ (Supplementary Fig. 1i, j). Differential expression analysis of high-throughput RNA sequencing data from whole *Drosophila* wing imaginal discs showed a high degree of overlap and correlation in changes of gene expression between the two *RpS3* alleles (Supplementary Fig. 1m, n and Supplementary Data 1), with 496 genes differentially expressed in both mutants compared to WT. This suggests that most of the differentially expressed genes are a consequence of the *RpS3* mutation itself, rather than resulting from different genetic backgrounds.

**Fig. 1:**
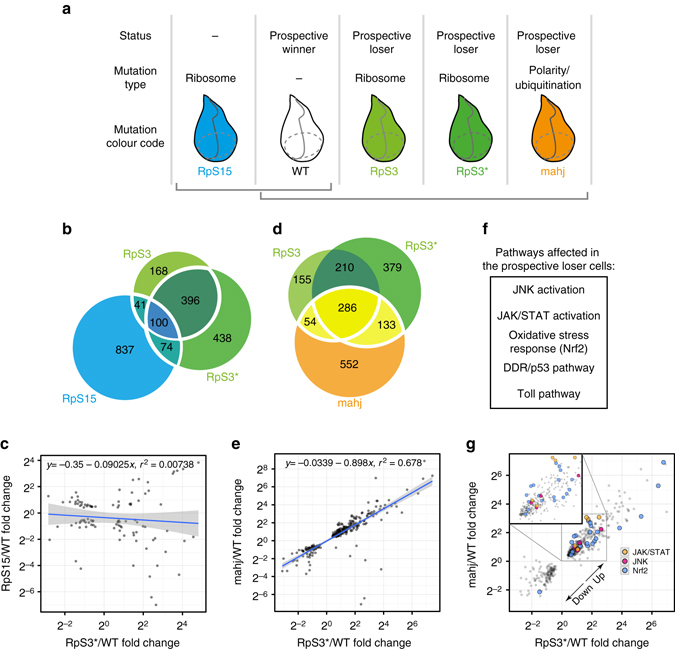
Transcriptional profiling identifies a molecular signature common to prospective loser cells. **a** Schematic representation of the genotypes used for transcriptional profiling and their corresponding prospective loser/winner status. **b** Venn diagram showing overlap of genes differentially expressed in *RpS3*, RpS3* and *RpS15* compared to wild type (WT). **c** Linear regression of fold changes of differentially expressed genes in WT vs. RpS3* and WT vs. *RpS15*, shaded area indicates 95% confidence interval for the fit. **d** Venn diagram showing overlap of genes differentially expressed in *RpS3*, RpS3* and *mahj* compared to WT. **e** Linear regression of fold changes of differentially expressed genes in WT vs. RpS3* and WT vs. *mahj*, shaded area indicates 95% confidence interval for the fit. **f** Pathways activated in prospective loser cells. **g** Data set of differentially expressed genes as in **e**, where genes from pathways in **f** are highlighted

Next we tried to establish whether the genes that are differentially expressed correlate with the propensity of a cell to behave as a loser. To this aim, we compared the *RpS3* ^*+/−*^ mutant transcriptomes to those of two additional mutants: *RpS15* ribosomal gene mutants (M(2)53), which exhibits the *Minute* phenotype^18^ but do not behave as losers against WT cells (Supplementary Fig. 1k, l); and mutants in *mahj*, a gene that is functionally unrelated to ribosomal genes (*mahj* is an interactor of polarity genes and a component of a cullin ubiquitin ligase complex^12, 40^), but whose mutation nevertheless confers a loser status against WT cells^12^ (Fig. 1a). Interestingly, there was little overlap between loser *RpS3* ^*+/−*^ and non-loser *RpS15* ^*+/−*^ cells (Fig. 1b, 100 genes vs. 60 genes overlap expected by random chance) and no significant correlation in expression level changes (Fig. 1c, *r* ^2^ ≈ 0). Strikingly, however, when we compared the list of genes differentially expressed between *mahj* ^*−/−*^ and WT (Supplementary Data 1) to the *RpS3* ^*+/−*^ mutant dataset we observed a remarkably large overlap (286 genes vs. 58 expected by random chance, Fig. 1d). Furthermore, changes in expression levels show a striking correlation across *mahj* ^*−/−*^ and the two *RpS3* ^*+/−*^ mutant data (Fig. 1e, *r* ^2^ ≈ 0.678 for mahj/RpS3* correlation). Thus, the transcriptional profile of *RpS3* ^*+/−*^ is more similar to that of an unrelated loser mutation than to that of a functionally related ribosomal mutation. This indicates that cells carrying the loser mutations *mahj* and *RpS3* share a common transcriptional molecular signature, suggesting an underlying similarity in their cellular states.

Given that such signature is associated with cells that behave as losers, we hypothesized that it could point towards genes and pathways potentially relevant for the acquisition of the loser status. Thus, we looked in more detail at the identity and function of the genes that compose this shared molecular signature. Specifically, we focused on the 443 differentially expressed genes that are shared between *mahj* ^*−/−*^ wing discs and at least one of the two *RpS3* ^*+/−*^ mutant datasets (Fig. 1d yellow intersection—excluding the small minority of genes whose changes in gene expression were in opposing directions—and Supplementary data 2). Gene Ontology (GO) analysis showed that this gene list is strongly enriched for genes associated with cellular stress response, oxidation–reduction processes and DNA repair (Supplementary Table 2). In addition, through manual annotation, we observed that prospective loser cells show altered expression in components of five key signalling pathways: the p53/DDR pathway, the Toll pathway, the JNK pathway, the JAK/STAT pathway and the oxidative stress response pathway (Fig. 1f). Furthermore, the majority of these genes were upregulated (Fig. 1g), suggesting that these pathways might be chronically activated in *RpS3* ^*+/−*^ and *mahj* ^*−/−*^ mutants.

First, we observed changes in the expression level of Toll pathway components, including pathway activators (*Traf4*, *cactin* and *Dif* (Supplementary Table 3)). This served as a proof of principle of the validity of our approach, as the Toll pathway has been shown to be activated downstream of cell competition and to lead to loser cells apoptosis^37^. Our data suggest that the Toll pathway might already be affected in prospective loser cells before cell competition.

Secondly, GO analysis showed that prospective loser cells activate several genes associated with the DNA damage response (DDR), such as *mre11*, *Lig4*, *ku80* and *lig3* (Supplementary Data 3). Since the DDR is often associated with p53 activation, we cross-compared our DDR signature genes with previously identified p53 targets in *Drosophila* that are upregulated following DNA damage^41, 42^. Indeed, we found that 29 of our DDR-related genes were also proposed p53 targets (Supplementary Fig. 1o, p). This suggests that the p53 pathway is activated in prospective losers. Interestingly, however, p53 activation in *Minute* cells has been shown not to be required for their autonomous cell survival or for their elimination during competition^39^.

Since the contribution of both p53 and the Toll pathway to cell competition has already been previously characterised, we did not pursue these pathways further. Instead we focused on characterising the involvement of JNK, JAK/STAT and the oxidative stress response pathways in cell competition.

### JNK signalling limits the expansion of loser cell clones

**Fig. 2:**
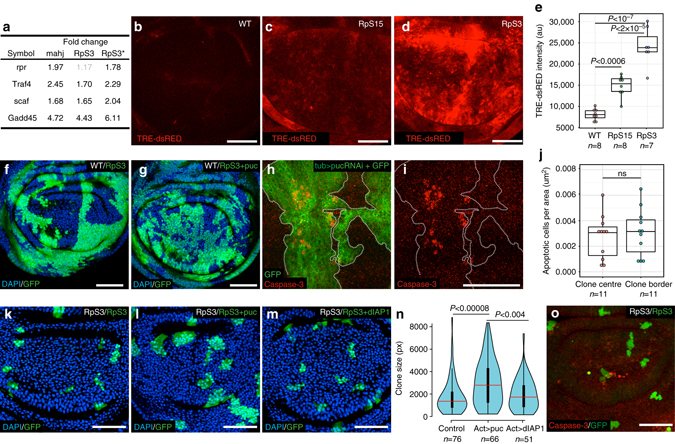
JNK pathway activity restricts the growth of prospective loser cells. **a** List of known JNK targets or regulators differentially expressed in the indicated genotypes (fold changes with false discovery rate>0.1 are in *grey*). **b**–**d** Activation of the transcriptional reporter of JNK activity (TRE16-dsRED, *red*) in WT (**b**), *RpS15* ^+*/−*^ (**c**) or *RpS3* ^+*/−*^ wing discs (**d**). **e** Quantification of fluorescence intensity from images as in **b**–**d** for the indicated genotypes, each dot represents a single wing disc. *P* values according to a post-hoc Tukey test. For all box and whisker plots the *horizontal line* represents the median and the upper/lower whiskers indicate the lowest/highest point within 1.5*interquartile range of the lower/higher quartile, respectively. **f**, **g** Wing disc harbouring 72-hour-old GFP-negative WT clones within GFP-positive *RpS3* ^+*/−*^ tissue with (**g**) or without (**f**) overexpression of *puc* only in the *RpS3* ^*+/−*^ cells (achieved using *upd3*-Gal4 to drive expression specifically in loser *RpS3* ^*+/−*^ cells). **h**, **i** Wing disc with clonal knockdown of *puc* (tub>puc-RNAi) and GFP expression (*green*) stained with anti-cleaved caspase-3, (*red*), clonal boundaries are indicated by a *dotted line*. **j** Quantification of the density of apoptotic cells in the centre of the clone or at the clonal boundary (i.e. within a 2-cell diameter of the clone periphery) for discs of the same genotype as in **h**; each dot represents a single wing disc. **k**–**m** *RpS3* ^*+/−*^ wing discs harbouring clones overexpressing GFP alone (**k**) or with the addition of *puc* (**l**) or dIAP1 (**m**). **n** Size distributions (in pixels) of clones of the same genotypes as in **k**, **m**, *P* values according to a post-hoc Tukey test. **o** *RpS3* ^*+/−*^ wing disc harbouring GFP-overexpressing clones (same genotype as in **k**) stained with anti-cleaved caspase-3. Detailed genotypes for each figure panel are listed in Supplementary Table 1. Scale bars, 50 μm throughout all figures

JNK (Basket (Bsk) in *Drosophila*) is a major stress response factor and an important regulator of cell growth and death in many *Drosophila* tissues^43–45^. JNK signalling has also been implicated in multiple cell competition systems, such as *scribble* ^2^, *mahj* ^12, 27^, *myc* ^28^, *APC* ^6^ and *Minute* competition^5, 23^, where it has been proposed to play a role in the late stage induction of cell death, although this is controversial in the case of Minute and myc-induced cell competition^29, 46^. Our RNAseq data indicates that both *Minute* and *mahj* ^*−/−*^ cells in their naive state upregulate the JNK pathway (as both JNK activators, such as *Gadd45* and JNK target genes, such as *scarface* ^47^ and *reaper* ^45^ are upregulated (Fig. 2a)), in agreement with earlier reports^27^. In addition we found that *RpS3* ^+*/−*^ cells show strong activation of the JNK reporter TRE-dsRED^48^ (Fig. 2b,d,e) and of active phosphorylated-JNK^6^ (pJNK; Supplementary Fig. 2a, c, d) compared to WT cells, whereas naive *RpS15* ^*+/−*^ cells, which do not behave as losers, show a markedly lower level of reporter activation (Fig. 2b,c,e and Supplementary Fig. 2a,b,d). The correlation between JNK activity and propensity to loser status prompted us to ask whether, in addition to the established late role in cell death, JNK signalling might play other roles in *Minute* cell competition. To address this, we inhibited JNK signalling specifically in loser *RpS3* ^+*/−*^ cells during cell competition, by overexpressing the JNK inhibitor *puckered (puc)* ^45, 49^. JNK inhibition reduced competition-induced cell death, as expected^23^ (Supplementary Fig. 2e–g). However, we also noted a striking increase in the overall ability of *RpS3* ^+*/−*^ cells to colonise the tissue (Fig. 2f, g), consistent with the possibility that JNK signalling might affect additional aspects of cell competition. We first asked whether JNK activation is required to prime cells for being killed by WT cells and tested whether high JNK signalling is sufficient to confer a loser status to otherwise WT cells. JNK overactivation leads to autonomous cell death^45^; however, after carefully optimising JNK activation levels (by partial silencing of the JNK inhibitor *puc* using *tub-Gal80* ^*TS*^, see Supplementary Table 1), we obtained large clones with active JNK, so as to assess whether they would behave as losers against surrounding WT cells. These clones displayed some residual apoptosis, however unlike during *Minute* cell competition, where cell death is observed within 1–2 cell diameters of the clone boundary^31^ (Supplementary Fig. 1i, j), there was no bias in the position of dying cells (Fig. 2h–j). Next we asked whether inhibiting JNK signalling in clones of *RpS3* ^*+/−*^ cells could boost their colonisation ability also under non-competitive conditions, when they are surrounded by other *RpS3* ^*+/−*^ cells. To this end, we generated GFP-positive clones overexpressing *puc* in an *RpS3* ^*+/−*^ background and scored clone size. Interestingly we observed again that *RpS3* ^*+/−*^ clones with reduced JNK activity grew larger than control *RpS3* ^*+/−*^ clones (Fig. 2k,l,n). Similar results were obtained expressing dominant-negative JNK (Bsk^DN^; Supplementary Fig. 3d–f), while JNK inhibition had no such effect on control WT clones (Supplementary Fig. 3a–c). This confirms that JNK has an impact on *Minute* cells beyond an involvement in competition-induced cell death. We next wondered whether the increase in clone size was due to the anti-apoptotic effect of JNK inhibition, which could inhibit background-level cell death in *Minute* cells^50^, leading to bigger clone sizes. This seemed unlikely because in our experimental conditions *RpS3* ^*+/−*^ wing discs display modest additional apoptosis (Fig. 2o and Supplementary Fig. 1c, d), or more generally cell death (Supplementary Fig. 2i–k), in the absence of cell competition. Consistent with this, inhibiting apoptosis by overexpressing dIAP1 (which effectively blocks damage-induced apoptosis; Supplementary Fig. 2h) had no effect on the size of naive *RpS3* ^*+/−*^ clones (Fig. 2k,m,n). Similarly, inhibiting apoptosis by drICE RNAi or by p35 overexpression had no significant effect on clone size (Supplementary Fig. 3g–l). Altogether this indicates that JNK signalling inhibition promotes the expansion of *RpS3* ^*+/−*^ clones beyond simply improving their survival.

**Fig. 3:**
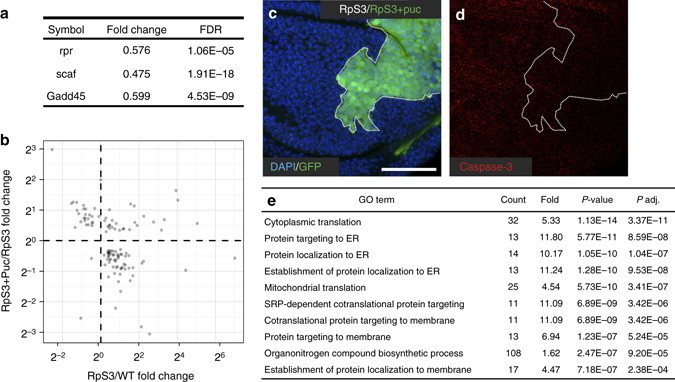
JNK inhibition partially reverts the molecular signature in *RpS3* ^*+/−*^ cells and increases the expression of translation-related genes. **a** List of known JNK targets that become downregulated on overexpression of the JNK-specific inhibitor *puckered* (*puc*) in *RpS3* ^*+/−*^ wing discs. **b** Plot showing fold changes in gene expression (*RpS3*+puc/*RpS3*, *y* axis; *RpS3*/WT, *x* axis) for those genes that are differentially expressed in *RpS3* ^*+/−*^ cells upon JNK inhibition vs. *RpS3* ^*+/−*^ cells and that are also present in the prospective loser signature. **c**, **d** *RpS3* ^*+/−*^ wing discs harbouring GFP-positive clones overexpressing *puc* (**c**) and stained with anti-cleaved caspase-3 (*red*, **d**). Note the absence of localised cell death at the clonal boundary (**d**). Detailed genotypes are listed in Supplementary Table 1. **e** Table of ten most highly enriched GO terms amongst genes that become upregulated on JNK inhibition in *RpS3* ^*+/−*^ cells with the number of genes (count), fold enrichment, *P* value and *P*-adj presented

To understand how JNK inhibition improves the growth potential of *RpS3* ^*+/−*^ cells, we carried out RNA-seq of *RpS3* ^*+/−*^ wing discs overexpressing *puc* and analysed at the whole transcriptome level what genes changed expression levels compared to reference *RpS3* ^*+/−*^ cells. We found that 2117 genes were differentially expressed (Supplementary Data 4) and, as a control, several JNK target genes that are upregulated in *RpS3* ^*+/−*^ discs were downregulated upon *puc* overexpression (Fig. 3a). We next compared how JNK inhibition affects the expression of the molecular signature genes common to prospective losers. Interestingly 26% (114 out of 443) of the molecular signature genes were differentially expressed upon Puc overexpression (Supplementary Data 5) and their expression levels anti-correlated with those of *RpS3* ^*+/−*^ discs, indicating a partial reversal of the molecular signature (Fig. 3b). However inhibiting JNK signalling in clones of *RpS3* ^*+/−*^ cells did not enable them to kill surrounding *RpS3* ^*+/−*^ cells (Fig. 3c, d). Thus, although JNK inhibition partially reverts the prospective losers molecular signature, its inhibition is not sufficient to turn *RpS3* ^*+/−*^ cells into winners against other *Minute* cells and therefore that does not explain why clones grow better. To get insight into the mechanism by which JNK signalling inhibition improves the growth of *Minute* clones we sought to identify by gene ontology analysis cellular pathways that are affected in prospective losers upon JNK inhibition. Strikingly, we found that amongst the most highly enriched pathways are many involved in protein translation and synthesis (Fig. 3e) with a large number of ribosomal genes upregulated in *RpS3* ^*+/−*^ cells upon JNK inhibition (Supplementary Fig. 3m). This suggests that JNK signalling inhibits the growth of *RpS3* ^*+/−*^ cells by downregulating their protein synthesis machinery. Altogether we conclude that JNK signalling has an active function in inhibiting loser cell growth and that, by restricting their intrinsic growth potential, it contributes to cell competition.

### Unpaired ligands produced by losers promote cell competition

**Fig. 4:**
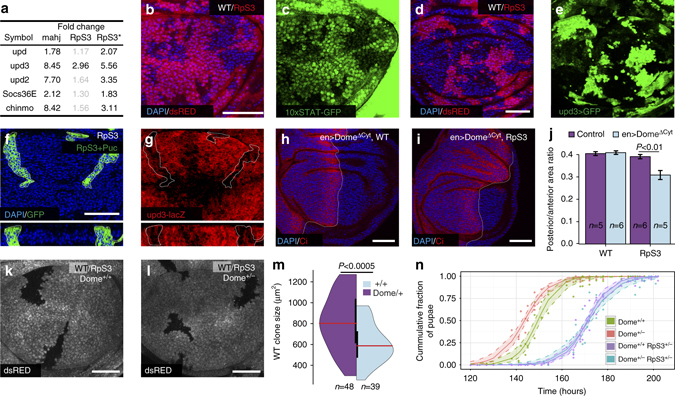
JAK/STAT activation is required for normal *RpS3* ^*+/−*^ tissue growth and for competition -induced overproliferation of winner cells. **a** List of known JAK/STAT targets and regulators differentially expressed in the indicated prospective loser genotypes (fold changes with false discovery rate>0.1 are in *grey*). **b**, **c** JAK/STAT reporter 10XSTAT-GFP expression in wing discs mosaic for *RpS3* ^+*/−*^ cells (ds-RED-positive) and WT cells (ds-RED-negative). **d**, **e** *upd3>*Gal4 reporter driving the expression of GFP in wing discs mosaic for *RpS3* ^+*/−*^ cells (ds-Red positive) and WT cells (ds-RED negative). **f**, **g** *upd3*-LacZ reporter activity (*red*, **g**) in *RpS3* ^*+/−*^ wing discs harbouring GFP-labelled clones (**f**) that overexpress *puc*. Both *xy* (*top*) and *xz* sections (*bottom*) of the same wing disc are presented with clones outlined (*white dotted line*). **h**, **i** Expression of dominant negative JAK/STAT receptor Dome (Dome^ΔCyt^) in the P compartment (labelled by absence of Ci (*red*)) of wild-type (WT) (**h**) or *RpS3* ^*+/−*^ wing discs (**i**). **j** Quantification of P/A compartment wing pouch ratios of WT or *RpS3* ^*+/−*^ wing discs expressing or not Dome^ΔCyt^ in the P compartment, as indicated; *P* values according to Wilcoxon rank sum test. **k**, **l** Wing disc harbouring WT clones (dsRED negative) in an *RpS3* ^*+/−*^ *Dome* ^*+/+*^ background (**k**) or in an *RpS3* ^+*/−*^ *Dome* ^+*/−*^ background (**l**). **m** Size distribution of WT clones induced in discs of genotypes as in **k**, **l**, as indicated, *P* values according to Wilcoxon rank sum test. **n** Developmental timing until pupariation represented as cumulative fraction of pupae over time for larvae of the indicated genotypes. Data were fitted to a sigmoid curve; the shaded area represents 99% confidence intervals for the fit. Each condition consists of at least five repeats of *n* > 17 larvae. Detailed genotypes for each figure panel are listed in Supplementary Table 1

The JAK/STAT pathway is a cytokine signalling pathway involved in the proliferative, immune and inflammatory responses^51, 52^. Specifically in *Drosophila* imaginal discs, the pathway is required for developmental patterning^53, 54^ and to promote proliferation under normal conditions and during regeneration^52, 55^. Our RNAseq data show that both *RpS3* ^*+/−*^ and *mahj* ^*−/−*^ cells upregulate the JAK/STAT targets *Socs36E* and *chinmo* as well as all three ligands of the pathway—the cytokines *unpaired 1, 2* and *3* (*upd*, *upd2*, *upd3*)^56–58^ (Fig. 4a), suggesting pathway activation. We first validated these findings using an in vivo fluorescent reporter of STAT activity (10xSTAT-GFP^59^). Indeed we found that *RpS3* ^*+/−*^ cells display reporter activation compared to WT cells (Figs. 4b, c) and upregulate *upd3* expression as detected by in situ (Supplementary Fig. 4a, b), by *upd3-Gal4, UAS-GFP* expression (Fig. 4d, e and Supplementary Fig. 4c, d), and by expression of *upd3*-LacZ^60^ (Supplementary Fig. 4e, f), confirming the RNAseq data. We next asked whether JNK signalling, which has been shown to activate Upd ligands in this and other tissues^60–62^, was responsible for the production of Upd ligands in *RpS3* ^*+/−*^ wing discs. Thus, we inhibited the JNK pathway in *RpS3* ^*+/−*^ wing discs and assessed the levels of *upd3* expression and JAK/STAT activity. Indeed JNK inhibition resulted in a reduction of JAK/STAT reporter levels (Supplementary Fig. 4i, j) and of *upd3*-LacZ expression (Fig. 4f, g, compare to control clones in Supplementary Fig. 4g, h), indicating that the JNK pathway contributes to JAK/STAT ligands production in *Minute* cells.

Next we assessed the physiological significance of JAK/STAT pathway activation for *RpS3* ^*+/−*^ wing discs. Notably JAK-STAT inhibition in the P compartment, by expression of a dominant-negative version of the receptor Dome (Dome^ΔCyt^)^63^ (whose activity we confirmed by monitoring the activity of the 10xSTAT-GFP reporter; Supplementary Fig. 4k, l) caused substantial growth inhibition and a markedly reduced P compartment size in *RpS3* ^*+/−*^ mutants, while it had no noticeable effect on WT cells (Figs. 4h–j). This indicates that *RpS3* ^*+/−*^ cells are more reliant on JAK/STAT activity for their growth than WT cells.

It has long been known that during cell competition winner cells proliferate faster than they would normally do on their own^5, 28–31^. This has mostly been attributed to winner–loser recognition signals or to apoptosis-induced proliferation^32–34^. However, Upds are soluble proteins capable of long-range signalling and have been reported to cause non-autonomous overgrowth phenotypes^61, 62^. In addition we recently showed that in the adult *Drosophila* midgut Upd ligands produced by *RpS3* ^*+/−*^ cells promote the proliferation of WT cells during cell competition^5^. This suggested that their production from *RpS3* ^*+/−*^ wing disc cells could have a similar effect on winner cell proliferation in this tissue. Indeed, we found that just removing one copy of the pathway receptor *Dome* was sufficient to inhibit substantially the growth of WT clones during cell competition in wing discs, indicating that winner cells depend on JAK/STAT signalling for competition-induced proliferation (Fig. 4k–m). Importantly, *Dome* heterozygosity did not cause any developmental delay in either WT or *Minute* animals (Fig. 4n), indicating that this effect is not simply due to a general inhibition of tissue growth or proliferation. We conclude that Upd ligands produced by *Minute* cells play a dual role: they promote growth in *Minute* tissues, however during cell competition they are also exploited by WT cells to boost their own proliferation.

### Prospective losers activate the oxidative stress response

**Fig. 5:**
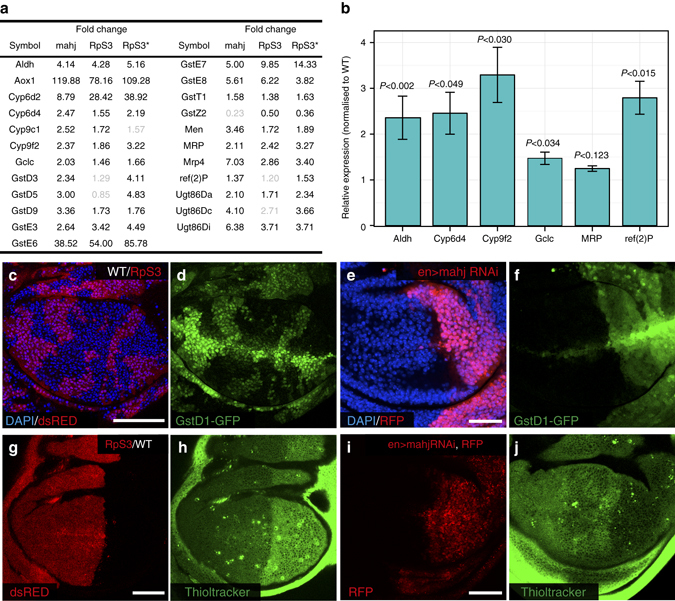
Prospective loser cells activate the oxidative stress response before and during competition. **a** List of genes involved in the oxidative stress response upregulated in the indicated prospective loser genotypes (fold changes with false discovery rate >0.1 are in *grey*). **b** Quantitative PCR analysis showing for selected genes from the list in **a** the expression levels in wing discs overexpressing Nrf2 relative to wild-type (WT), *P* values according to Welch *t*-test; error bars: s.e.m. **c**, **d** Expression of the Nrf2 reporter *GstD1*-GFP (*green*, **d**) in wing discs mosaic for *RpS3* ^+*/−*^ cells (ds-RED-positive) and WT cells (ds-RED-negative, **c**). **e**, **f** *GstD1*-GFP reporter expression (*green*, **f**) in wing discs expressing *mahj* RNAi in the P compartment (RFP-positive; **e**). **g**, **h** Thioltracker staining (*green*, **h**) in composite wing discs carrying a (ds-RED-positive) *RpS3* ^*+/−*^ A compartment and a WT (ds-RED-negative) P compartment (**g**). **i**, **j** Thioltracker staining (*green*, **j**) in wing discs expressing *mahj* RNAi in the P compartment (RFP-positive; **i**). Thioltracker levels are lower in the *mahj* RNAi compartment in 10 out of 18 wing discs analysed (56% penetrance). Detailed genotypes for each figure panel are listed in Supplementary Table 1

One of the most prominent features of the molecular signature common to prospective loser cells is the marked upregulation of many (23) genes involved in oxidation/reduction and detoxification processes (Fig. 5a). These include multiple known target genes of the mammalian transcription factor Nrf2^64^ (*CncC* in *Drosophila* ^65^), although neither the *CncC* gene itself nor the canonical inhibitor of the pathway *Keap1* are transcriptionally affected (Supplementary Data 1). Nrf2 is the master regulator of the oxidative stress response and is involved in the adaptation to oxidative and chemical stress through upregulation of a number of genes with anti-oxidant function or involved in the removal of harmful oxidation products^64^. Specifically, we found that prospective loser cells show upregulation of genes involved in glutathione synthesis (glutamate-cysteine ligase catalytic subunit—Gclc), glutathione conjugation to xenobiotics (glutathione S-transferases—GSTs), xenobiotic export (multidrug-resistance like proteins), xenobiotic glucuronidation (UDP-glucose-glycoprotein glucosyltransferases) and oxidation (P450 family cytochromes—Cyp) (Fig. 5a). While many of these genes are predicted Nrf2 targets based on mammalian studies, this had not been validated in *Drosophila*. To assess whether activation of these genes is indicative of Nrf2 activity we asked, for some of the genes in Fig. 5a, whether they would be upregulated upon Nrf2 overexpression. Indeed Nrf2 overexpression caused upregulation of 5/6 genes that we tested (Fig. 5b; 1/6 is only marginally upregulated), consistent with Nrf2 activation in prospective losers. To confirm activation of the Nrf2 pathway in loser cells, we used a fluorescent transcriptional reporter of Nrf2—*GstD1-*GFP^65^. In line with our RNAseq data, both *RpS3* ^*+/−*^ (Fig. 5c, d and Supplementary Fig. 5a–c) and *mahj* RNAi (Fig. 5e, f) cells exhibited higher levels of reporter expression compared to WT cells, both before and during cell competition, confirming pathway activation.

We next wondered what oxidative insult might be causing activation of the Nrf2 pathway. Reactive oxygen species (ROS), are powerful activators of Nrf2 and have been shown to be produced under some metabolic imbalance conditions^61^. However, *RpS3* ^*+/−*^ cells showed only a negligible increase in dihydroethidium (DHE) staining (Supplementary Fig. 5d, e) and a decrease in CM-H_2_DCFDA staining (Supplementary Fig. 5f, g), two widely used fluorescent dyes sensitive to different types of ROS^61^. Given that prospective loser cells display the upregulation of several enzymes involved in glutathione synthesis or utilisation (Fig. 5a) and given that free reduced glutathione is the main cytosolic anti-oxidant and regulator of the cytosolic redox state^66^, we wondered whether the oxidative stress response might instead be triggered by low cytosolic levels of reduced glutathione. Notably, using ThiolTracker Violet, a fluorescent sensor of intracellular free thiols, we found that both *RpS3* ^*+/−*^ and *mahj* RNAi cells show lower levels of reduced glutathione than WT cells (Fig. 5g–j and Supplementary Fig. 5h). Thus an oxidative cytosolic environment is a hallmark of cells with reduced competitive ability and may contribute to activation of the Nrf2 pathway.

**Fig. 6:**
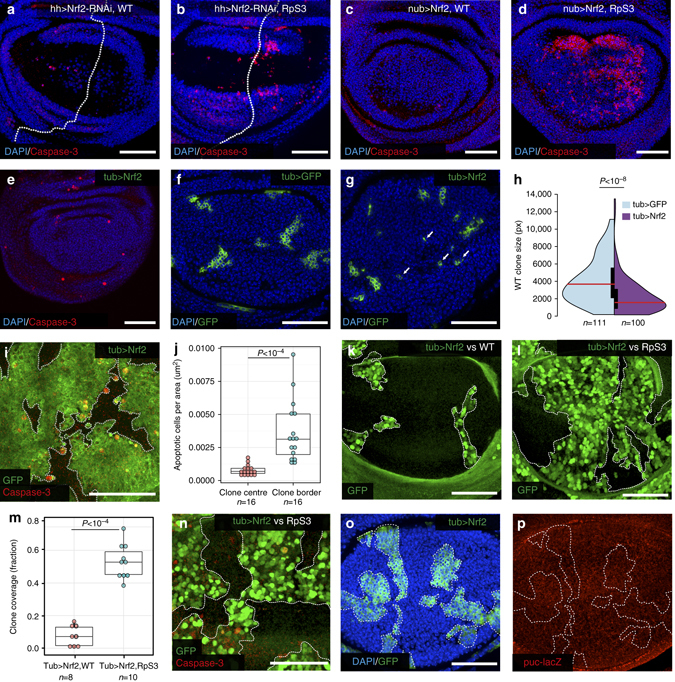
Nrf2 activation is required for *RpS3* ^*+/−*^ cell survival and is sufficient to confer the loser status. **a**, **b** WT (**a**) or *RpS3* ^*+/−*^ (**b**) wing discs with knockdown of Nrf2 in the P compartment and stained with an anti-cleaved Caspase-3 antibody. Dotted line indicates the A/P boundary. **c**, **d** WT (**c**) or *RpS3* ^*+/−*^ (**d**) wing discs overexpressing Nrf2 in the pouch, stained with an anti-cleaved Caspase-3 antibody. **e** Wing disc mildly overexpressing Nrf2 throughout (see genotype in methods), stained with an anti-cleaved Caspase-3 antibody. Note the absence of significant autonomous cell death (cleaved Caspase-3, *red*). **f**, **g** Wing discs harbouring clones overexpressing GFP alone (**f**) or with the addition of Nrf2 (**g**) (arrows indicate instances of clone fragmentation). **h** Size distributions represented as split-violin plots of clones from genotypes as in **f**, **g**. *P*-values calculated using Wilcoxon rank sum test. **i** Wing discs harbouring clones overexpressing GFP and Nrf2 and stained with an anti-cleaved Caspase-3 antibody (*red*). **j** Quantification of the density of apoptotic cells in the clone centre or at the clone boundaries, i.e., within a 2-cell diameter of the clone periphery, for discs of the same genotype as in **i**; each dot represents a single wing disc. *P* values calculated using paired sample *t*-test. **k**, **l** wing discs with GFP-labelled Nrf2 overexpressing clones (otherwise wild-type) surrounded by WT (**k**) or by *RpS3* ^*+/−*^ (**l**) cells (see genotypes in methods). **m** Quantification of GFP-positive clone area as a fraction of the entire pouch region in wing discs from **k**, **l**. Each dot represents one wing disc and the *P* value is according to the Welch *t*-test. **n** Wing disc with GFP-labelled Nrf2 overexpressing clones (otherwise WT) surrounded by *RpS3* ^*+/−*^ cells as in **l** and stained with anti-cleaved Caspase-3 (*red*). Note the absence of cell death at clonal boundaries (outlined with *white dotted line*). **o**, **p** Activity of the *puc*-LacZ reporter (*red*, **p**) in WT wing discs harbouring GFP-labelled clones that overexpress Nrf2 (*green*, **o**) at same levels as in **i**. Detailed genotypes for each figure panel are listed in Supplementary Table 1

Nrf2 is generally considered a stress adaptation factor and typically serves a pro-survival role^64, 65^. Indeed we found that knockdown of Nrf2 by Nrf2 RNAi causes increased cell death in *RpS3* ^*+/−*^ cells, but not in WT cells (Fig. 6a, b and Supplementary Fig. 6a), indicating that *RpS3* ^*+/−*^ cells are more reliant on Nrf2 than WT cells for their survival. We therefore hypothesized that increasing Nrf2 activity might be beneficial for loser cells and rescue them from elimination. Unexpectedly, strong overexpression of Nrf2 with *hh-Gal4* caused widespread cell death in both *RpS3* ^*+/−*^ cells (Supplementary Fig. 6c) and WT cells (Supplementary Fig. 6b), which we found to be JNK-dependent (Supplementary Fig. 6d, e). However milder Nrf2 overexpression, by using *nubbin-Gal4*, a weaker Gal4 driver, resulted in widespread *RpS3* ^*+/−*^ cell lethality while only mildly affecting WT wing discs (Fig. 6c, d). Thus, while *RpS3* ^*+/−*^ cells rely on Nrf2 function, they are also more sensitive to its overactivation, possibly because this adds onto already high Nrf2 activity, reaching lethal levels.

Given that activation of the Nrf2 pathway is a hallmark of loser cells and that high levels can lead to autonomous cell death, we wondered whether it might contribute to the loser status. We therefore decided to ask whether Nrf2 activation can turn WT cells into losers. To this aim we needed to identify a Gal4 driver that would allow clonal overexpression of Nrf2 at levels that were insufficient to cause cell-autonomous death. Using *tub>*CD2*>Gal4* in combination with *tub-Gal80* ^*TS*^, we identified suitable temperature conditions in which entire wing discs expressing Nrf2 (by germline excision of the CD2 cassette) are capable of normal development and show little apoptosis (Fig. 6e). However, using exactly the same Gal4 driver and temperature conditions, we observed that Nrf2 expressing clones were significantly smaller than control GFP-expressing clones (Fig. 6f–h), with visible signs of cell fragmentation, indicative of cell death (Fig. 6g, arrows). This suggested that Nrf2 overexpressing clones could be eliminated through cell competition. To test this, we generated larger Nrf2 overexpressing clones, so that within the same Nrf2 overexpressing clones we could score for differences in the frequency of dying cells between the centre of the clone and the clone border, where cell competition is supposed to take place. Notably, we found that the vast majority of apoptotic cells localised at the periphery of Nrf2 clones (Fig. 6i, j). In addition, WT clones overexpressing Nrf2 grew much better when surrounded by *Minute RpS3* ^*+/−*^ cells than when surrounded by WT cells (Fig. 6k–m) and showed no accumulation of death at clonal boundaries (Fig. 6n). Thus both clonal growth and boundary death are context-dependent and result from the interaction of Nrf2 overexpressing cells with WT cells. Furthermore, Nrf2 overexpressing cells interfaced with WT cells at the anterior-posterior compartment boundary, which is known to pose a barrier to cell competition, were protected from death (Supplementary Fig. 6f, g). Together this shows that Nrf2-overexpressing cells are eliminated by cell competition. Notably, we found that Nrf2 overexpression does not cause JNK activation (Fig. 6o, p and Supplementary Fig. 6h, i), indicating that Nrf2 is not upstream of constitutive JNK activation in *Minute* cells and that JNK activation is not necessary for cells to acquire the loser status. We conclude that Nrf2 activation alone, which we found to be a hallmark of prospective loser cells, is sufficient to prime cells for their elimination by WT cells and convert them into losers.

## Discussion

**Fig. 7:**
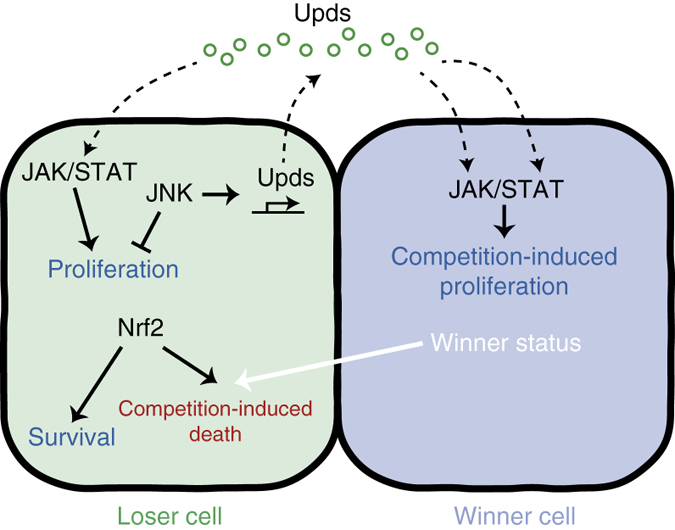
A proposed model for the contribution of JNK, JAK/STAT and Nrf2 pathways to cell competition. JNK signalling activation restricts the growth of loser cells. JAK/STAT signalling, activated by the Unpaired ligands secreted by the loser cells, plays a dual role: it promotes the proliferation of prospective loser tissue, as well as the overproliferation of winner cells during cell competition. Nrf2 pathway activation in losers also plays a dual role: it promotes prospective loser cell survival; however it also primes loser cells for their elimination by WT neighbours during cell competition

Despite substantial recent progress, our understanding of the mechanisms by which cells compete is still sketchy. While it is increasingly recognised that cells can compete via multiple unrelated mechanisms^13, 23–25, 36–38, 67, 68^ our understanding of even the best characterised and prototypical models of cell competition, such as *Minute* cell competition, is still rather fragmented. In this work, we provide the first comparative analysis of transcriptomes from prospective loser cells carrying functionally unrelated loser mutations. Our analysis highlights a remarkable degree of similarity in the gene expression profiles of two independent prospective loser populations, *RpS3* ^*+/−*^ and *mahj* ^*−/−*^, revealing that some prospective loser cells can share a common molecular signature and, as we show, a common route to cell competition. A significant component of this signature is the shared activation of multiple stress response pathways, which indicates that these loser populations share a common cellular state. Functional analysis of the prospective loser cell signature revealed that both *RpS3* ^*+/−*^ and *mahj* ^*−/−*^ cells constitutively activate JNK, JAK/STAT and oxidative stress response pathways. As we show, each acts as a molecularly separable module to control distinct aspects of cell competition: loser cell proliferation, winner cells overgrowth and loser cell elimination, respectively (Fig. 7). These findings advance our understanding of the mechanisms underpinning this process but also highlight the complexity of the range of cell signalling and cell–cell interactions that collectively contribute to cell competition. They further indicate that our dataset of 443 prospective loser signature genes, the majority of which is still uncharacterised for their contribution to cell competition, is a valuable resource that may offer additional fundamental insights on the mechanisms of cell competition.

Constitutive JNK pathway activation in loser cells has been previously reported^27^. However, it was thought that JNK signalling simply provides a pro-apoptotic signal during competition. Our data indicate that instead JNK activation plays an additional role and contributes to cell competition by restricting the growth potential and clonal expansion of loser cells. Thus, the notoriously slow proliferation rate of *Minute* cells is not just a consequence of their intrinsically limiting translational capacity, but is also actively imposed on them by JNK activation.

Several reports have indicated that winner cells enhance their proliferation in various cell competition models^5, 6, 8, 28–31^, including *Minute* competition^5, 29, 31^. However, it has more recently been reported that during *Minute* cell competition WT cells grow at their normal cell-autonomous rates^69^. This is in apparent contradiction with our finding that during cell competition *Minute* cells promote the proliferation of WT cells above their intrinsic rate by stimulating JAK/STAT signalling. We think that this apparent discrepancy can be resolved, if considering the experimental design in Martin and Morata^69^. Their conclusion was based on the observation that clones of WT cells, allowed to grow for the same amount of time (e.g., 48 h) before they are analysed at the end of third instar, reach similar sizes irrespective of whether they were in a WT or in a *Minute* host. However, considering that *Minute* larvae are developmentally delayed, effectively this equates to inducing clones at different developmental stages in the two genetic backgrounds, with WT clones being ‘developmentally older’ when induced in *Minute* larvae. Since the proliferation rate in wing discs progressively slows down, older cells would be expected to generate smaller clones; we suggest that the fact that they do not generate smaller clones is rather an indication that WT cells are indeed proliferating faster than normal, in line with our findings. Furthermore our results are consistent with our earlier observation that in the adult posterior midgut WT clones overgrow during *Minute* cell competition in a JAK-STAT-dependent manner^5^. This might have been a peculiarity of the midgut, where JAK-STAT signalling is commonly activated as part of the gut homeostasis/repair^70^. Our new data indicate instead that this is a general phenomenon integral to *Minute* cell competition.

These findings address a long-standing question in the field and provide a mechanism to explain how an important hallmark of cell competition—the overproliferation of winner cells—is induced. Indeed, while some of the signals involved in loser cell elimination have now been identified^23, 32, 36–38, 71^, no signal involved in the overproliferation of fitter cells during cell competition had been found so far. It has been suggested that winner cells overproliferation stems from winner/loser recognition^32, 33^, or from compensatory proliferative signals emanating from dying loser cells^31, 34^. Our data instead demonstrates that, at least for *Minute* cell competition, the overproliferation of winner cells is not a consequence of and in fact is independent of winner/loser cell recognition and of loser cell death, since Upd-2 and Upd-3 production is activated across *Minute* tissues in a JNK-dependent manner prior to and independently of cell competition.

Interestingly, it has previously been shown that high STAT activity can induce the winner status^24^. Thus it seems counterintuitive that *Minute* cells, which have high stat levels (most likely as a result of autocrine signalling), are losers against WT cells with lower STAT signalling. This indicates that despite having enhanced JAK/STAT signalling, *Minute* cells are impaired in their ability to become winners due to their underlying ribosomal mutation. In other words, *Minute* acts epistatically to their JAK/STAT activation. Similar interactions between pathways that define fitness have been reported previously. For example cells that overexpress dMyc, lose their ability to outcompete cells if they are also *Minute* ^*+/−*^^28^.

In the economy of optimising tissue health, the dual survival/competition role of JAK/STAT signalling is rather sensible: the same signal that allows *Minute* cells to boost their own proliferation to compensate for defective growth ensures also their out-competition by fitter cells when these are present. In addition this observation might help explain the elevated cancer risk associated with patients affected by ribosomopathies^72^, genetic diseases caused by mutations in ribosome genes. Given that inflammation plays a key role in promoting cancer^73^ our findings that cells with ribosomal mutations activate a chronic inflammatory/pro-proliferative response could provide a molecular explanation for this predisposition.

One of the most interesting aspects of cell competition is the selective induction of apoptosis in the loser cell population. Exciting recent advances point at a role for Flower, Toll and PTP10D signalling as factors involved earmarking cells as losers and/or in their elimination^32, 37, 38^. However what pre-existing conditions prime cells as losers and initiate cell competition is not known. Importantly, our work reveals that *RpS3* ^*+/−*^ and *mahj* ^*−/−*^ cells share a common chronic activation of the oxidative stress response and that this response alone (by Nrf2 overexpression) is sufficient to turn otherwise WT cells into losers. Together these findings suggest that *RpS3* ^*+/−*^ and *mahj* ^*−/−*^ cells are marked as loser cells by chronic activation of the Nrf2 pathway. Although the mechanism underlying Nrf2 pathway activation in prospective loser cells remains unclear, our Nrf2 overexpression experiments indicate that we can generate losers in the absence of actual oxidative damage. These findings suggest that the response to damage rather than damage itself turns cells into losers. Furthermore, Nrf2 activation does not lead to constitutive JNK activation, further supporting our conclusion that JNK activity, despite showing widespread activation in losers, is not involved in earmarking cells for elimination.

Nrf2 canonically serves a pro-survival role, protecting cells from the negative effects of toxic/oxidising compounds^64, 65^. This is the case also for *RpS3* ^*+/−*^ and *mahj* ^*−/−*^ cells, since silencing Nrf2 causes autonomous cell death in *Minute* cells. Thus, we propose that the Nrf2 pathway, has a dual survival/competition role in *Minute* cells: it is required for their viability, but it is also responsible for priming cells as losers in the presence of fitter cells.

From an organismal perspective, since cells undergoing oxidative stress are likely to accumulate damage over time, it might be beneficial to remove these stressed cells and replace them with healthy neighbours. As oxidative stress is activated in a number of pathological conditions including cancer^74^, our findings suggest that cell competition might be common in these instances and potentially plays a role in disease prevention and progression. How the Nrf2 pathway primes cells as losers and how this might impact on diseases such as cancer are important new questions that remain to be addressed.

## Methods

### Fly maintenance and clone induction

All flies were raised at 25 °C on a standard wheat flour-based food supplemented with yeast. For experiments, eggs were collected for 24 h and larvae were dissected at wandering third instar stage. Clones were induced using heat-shock inducible flippase (FLP) recombinase (at 37 °C, in a water bath) either through mitotic recombination or by excision of a cassette flanked by FRT sites (as indicated in figure legends). Heat shock conditions, i.e., duration of heat shock (5–60 min) and clone age at dissection (48–72 h), were optimised separately for each experiment and are listed in Supplementary Table 1. For experiments using the Gal80^TS^ system, flies were raised at 28–28.5 °C (Nrf2 overexpression) or 27 °C (puc-RNAi) following clone induction.

### *Drosophila* stocks

Genotypes used for each experiment are detailed in Supplementary Table 1. The following *Drosophila* stocks were used: *RpS3*[Plac92] (BL5627), RpS3*(BL5699), *M(2)53* ^1^ (BL5698), FRT42D *mahj* ^12^, *TRE-16* ^48^, UAS-*puc-RNAi* (B. Edgar), w+/w-; tub>CD2>Gal4, UAS-GFP; tub-Gal80^TS^ (B. Edgar), UAS-*Puc* ^14C^ (E. M. Blanco), UAS-p35, *upd3-Gal4*, UAS-*GFP* (N. Perrimon), *10xSTAT-GFP* ^59^, UAS-*Dome* ^*ΔCy*t^ ^63^, *Dome* ^*g0218*^ *(BL11953), GstD1-*GFP^65^, UAS-*mahj-RNAi (*BL34912), UAS-Nrf2-RNAi^65^, UAS-*Nrf2* ^65^, UAS-*dIAP1* (P. Meier), *upd3*-LacZ^60^ (D. Bilder), UAS-Bsk^DN^ (E. Martin Blanco), puc[A251]-lacZ (BL11173) and UAS-DrICE RNAi (NIG HMS00398).

### Immunofluorescence

Late third instar larvae were dissected in phosphate-buffered saline (PBS) before fixing in 4% formaldehyde/PBS solution for 20 min. Dissected larvae were then washed in PBS and permeabilised in 0.25% Triton X-100 in PBS (PBS-T) for 20 min and blocked in 4% fetal calf serum in PBS-T (blocking buffer) for 30 min. Samples were incubated in primary antibody (diluted in blocking buffer) overnight at 4 °C and were subsequently washed three times (10 min each) in PBS-T. They were then incubated with secondary antibodies diluted in blocking buffer for 1 h followed by three further washes in PBS-T. All steps were performed on a rocking platform at room temperature unless otherwise indicated. Wing discs were mounted in Vectashield (Vector laboratories) using a borosilicate glass slide (no 1.5, VWR International). The following primary antibodies were used: rabbit anti-pJNK pTPpY (1:500, Promega V793B) 1/500, chicken anti-beta-galactosidase (1:1000, Abcam ab9361), rabbit anti-cleaved caspase 3 (1:500, Cell Signalling 9661 or 1:25,000, Abcam 13847), rat anti-Ci (1:1000, DSHB 2A1), chicken anti-GFP (1:1000, Abcam 13970) and sheep anti-Digoxigenin-AP (1:2000, Roche 11093274910). The secondary antibodies used were conjugated with Alexa 488, Alexa 555 or Alexa 633 dyes (Molecular probes). Nuclei were counterstained with DAPI (0.5 μg ml^−1^).

### RNA in situ hybridisation

All solutions were diluted in diethylpyrocarbonate (DEPC)-treated water. Third instar larvae were dissected in PBS, fixed in 4% paraformaldehyde/PBS for 20 min, washed first in PBS, then in PBS, supplemented with tRNA (250 ug ml^−1^) and heparin (50 ug ml^−1^). Antisense DIG-labelled upd3 RNA probe was prepared from linearised plasmid (DGRC clone FI03911) and hybridised overnight at 60 °C in a buffer containing deionised formamide (50%), SSC (5×), heparin (250 ug ml^−1^), tween (0.1%), heat-denaturated salmon-sperm DNA (100 ug ml^−1^) in DEPC water. Samples were then washed in high-stringency at 60 °C, in three dilutions (1:4; 1:1; 4:1, vol/vol) of buffers of PBS, tween 0.1% (PBT) and hybridisation buffer. Samples were then washed in PBT at room temperature, incubated with sheep anti-DIG conjugated to alkaline phosphatase (1/2000, Roche 11093274910), washed in PBT and processed for reaction with NBT/BCIP (Roche 11681451001). After reaction and washes in PBT, larvae were post-fixed and immunostained for GFP and mounted as described above. Discs were imaged on a Carl Zeiss Axioplan 2 with the Volocity software.

### Glutathione detection

All incubations were carried out in a PBS solution supplemented with: 50 mM d-Trehalose (Sigma T9531), 0.4 mM d-Glucose (Sigma G8270), 5 mM CaCl2, 15 mM MgSO_4_, 12.3 mM Glutamine. Late third instar larvae were dissected and incubated for 15 min with 5 μM ThiolTracker Violet (Molecular Probes T10095) at room temperature. Dissected larvae were then washed three times for 1 min before being mounted in the same solution on a glass slide. Slides were imaged immediately on a confocal microscope.

### ROS detection using DHE staining

Larvae were dissected in Schneider’s medium (Life Technologies) and stained with 4 μM DHE (Life Technologies) diluted in Schneider’s medium for 15 min. Subsequently the samples were washed twice in Schneider’s medium for 2 min, once in PBS for 5 min, fixed in 4% formaldehyde in PBS solution for 20 min, mounted in PBS and imaged immediately afterwards on a confocal microscope.

### CM-H_2_DCFDA staining

Larvae were dissected in Schneider’s medium (Life Technologies) and stained with 2 mM CM-H_2_DCFDA (Life Technologies) diluted in Schneider’s medium for 10 min. Subsequently the samples were washed three times in Schneider’s medium (2 min each), mounted in Schneider’s medium and imaged immediately afterwards on a confocal microscope.

### Propidium iodide staining

Hemi-dissected larvae were incubated in PBS supplemented with 10 μg ml^−1^ propidium iodide for 5 min at room temperature before fixation in 4% paraformaldehyde for 20 min at room temperature. Wing discs were then dissected and mounted as described in the immunofluorescence protocol above.

### Image acquisition and processing

All images were acquired on a Leica SP5 inverted or Leica SP8 upright confocal microscope, using either a 40 × 1.3 NA PL Apo or 40×/1.3 HC PL Apo CS2 Oil objective, respectively. All wing discs were imaged as z-stacks with each section corresponding to 1 μm. Clone areas were measured on a medial section of the pouch region either manually or using a custom script in Fiji^75^. For cell death quantifications, cells that were positive for cleaved caspase-3 (by immunostaining) and within the pouch region were counted. The total number of dying cells was normalised to the respective clone area/perimeter. Caspase-positive cells within a 2-cell diameter of the periphery of a clone were classed as ‘clone border’, whereas those further within were classed as ‘clone centre’. Both counts were then normalised to their respective areas. Images were analysed and processed using Fiji (version 2) and Photoshop (Adobe version CS6).

### RNAseq and quantitative PCR

For details on RNAseq data generation and analysis of the data on quantitative PCR please see Supplementary Methods.

### Statistical tests

Statistical tests were performed using R programming language. *P* values were determined using either a paired sample *t*-test, a Welch *t*-test, a post-hoc Tukey test or the Wilcoxon rank sum test (see figure legends). A minimum of three biological replicates were used for each experiment. For all box and whisker plots the horizontal line represents the median and the upper/lower whiskers indicate the lowest/highest point within 1.5*interquartile range of the lower/higher quartile, respectively.

### Developmental timing

Embryos of the indicated genotypes were collected at 3 h intervals and placed on standard wheat flour-based food supplemented with yeast at 25 °C. Emerging pupae were scored over time. Each genotypic condition was scored in 5–7 independent repeats. The number of pupae was normalised to the total amount of pupae per vial and plotted as the cumulative fraction of pupariating larvae per time. The data points were fitted with a sigmoid function using a nonlinear least squares method. The respective confidence intervals were calculated using a Monte Carlo simulation method (available from R package ‘propagate’).

### Data availability

The authors declare that all data supporting the findings of this study are available within the article and its Supplementary Information files or from the corresponding author upon reasonable request. RNA-seq data have been deposited in the National Center for Biotechnology Information GEO database under the accession code GSE92431.

## Electronic supplementary material

Supplementary Information

Supplementary Data 1

Supplementary Data 2

Supplementary Data 3

Supplementary Data 4

Supplementary Data 5
