# Supplementary material for: Chronic activation of JNK JAK/STAT and oxidative stress signalling causes the loser cell status
Source: Nat Commun. 2017 Jul 26;8:136. doi: 10.1038/s41467-017-00145-y (PMC5526992; doi:10.1038/s41467-017-00145-y)
Supplement: Supplementary file 1 — Supplementary Information [file 41467_2017_145_MOESM1_ESM.pdf]

Title of file for HTML: Supplementary Information
Description: Supplementary Figures, Supplementary Tables, Supplementary Methods, Supplementary References.

Title of file for HTML: Supplementary Data 1
Description: **List of genes differentially expressed by RNAseq between prospective loser cells and WT.** Sheets contain genes differentially expressed between WT and *mahj*$^{-/-}$ (mahjDE sheet), WT and *RpS3*$^{+/-}$ (RpS3DE sheet), or WT and *RpS3**$^{+/-}$ (RpS3starDE sheet). Columns contain the following information (as indicated at the top): Flybase gene ID, mean expression (of normalized read counts across all samples), WT expression (mean of normalised read counts); mutant expression (mean of normalised read counts); fold change (change in expression levels between respective mutant and WT tissues); log2(fold change) (logarithm to base 2 of the fold change); p-value (p-value for fold changes different from 0); FDR (p-value adjusted for multiple testing with the Benjamini-Hochberg procedure); symbol (Flybase gene symbol) and Fullname (Flybase full gene name).

Title of file for HTML: Supplementary Data 2
Description: **The prospective loser cell signature.** The list contains all genes that show significant differential expression in loser cells (i.e. *mahj* and at least one *Rps3* mutant) compared to WT in a correlated manner (i.e. consistently upregulated or consistently downregulated in losers). Columns contain the following information (as indicated at the top): Flybase ID, mahj fold change (relative expression of gene in *mahj*$^{-/-}$ cells versus WT), mahj FDR (p-value for mahj fold change adjusted for multiple testing with the Benjamini-Hochberg procedure), *RpS3* fold change (relative expression of gene in *RpS3*$^{+/-}$ cells versus WT), *RpS3* FDR (p-value for *RpS3* fold change adjusted for multiple testing with the Benjamini-Hochberg procedure), *RpS3** fold change (relative expression of gene in *RpS3**$^{+/-}$ cells versus WT), *RpS3** FDR (p-value for *RpS3** fold change adjusted for multiple testing with the Benjamini-Hochberg procedure), symbol (Flybase gene symbol) and Fullname (Flybase full gene name).

Title of file for HTML: Supplementary Data 3
Description: **Table of genes differentially expressed in prospective loser cells that relate to the p53/DNA Damage response pathway.** Columns contain the following information (as indicated at the top): Flybase gene ID, gene symbol (Flybase gene symbol), *mahj* fold change (change in expression levels between *mahj*$^{-/-}$ and WT tissues), *RpS3* fold change (change in expression levels between *RpS3*$^{+/-}$ and WT tissues), *RpS3** fold change (change in expression levels between *RpS3**$^{+/-}$ and WT tissues), *RpS3** FDR (p-value for *RpS3** fold change adjusted for multiple testing with the Benjamini-Hochberg procedure).

Title of file for HTML: Supplementary Data 4
Description: **List of genes differentially expressed by RNAseq between *RpS3* and *RpS3*+puc wing discs.** Sheets contain genes differentially expressed between *RpS3* mutant wing discs and *RpS3* mutant wing discs overexpressing the gene *puckered*. Columns contain the following information (as indicated at the top): Flybase gene ID, mean expression (of normalized read counts across all samples), *RpS3* expression (mean of normalised read counts); *RpS3*+puc expression (mean of normalised read counts);

fold change (change in expression levels between respective mutant and WT tissues); log2(fold change) (logarithm to base 2 of the fold change); p-value (p-value for fold changes different from 0); FDR (p-value adjusted for multiple testing with the Benjamini-Hochberg procedure); symbol (Flybase gene symbol) and Fullname (Flybase full gene name).

Title of file for HTML: Supplementary Data 5
Description: **Effects of JNK inhibition on the prospective loser signature.** The list contains the overlap between two datasets: genes within the molecular signature of loser cells and genes differentially expressed between *RpS3* and *RpS3*+puc cells. Columns contain the following information: Flybase ID, mahj fold change (relative expression of gene in *mahj*$^{-/-}$ cells versus WT), mahj FDR (p-value for mahj fold change adjusted for multiple testing with the Benjamini-Hochberg procedure), *RpS3* fold change (relative expression of gene in *RpS3*$^{+/-}$ cells versus WT), *RpS3* FDR (p-value for *RpS3* fold change adjusted for multiple testing with the Benjamini-Hochberg procedure), *RpS3** fold change (relative expression of gene in *RpS3**$^{+/-}$ cells versus WT), *RpS3** FDR (p-value for *RpS3** fold change adjusted for multiple testing with the Benjamini-Hochberg procedure), *RpS3*+puc fold change (relative expression of gene in *RpS3*+puc versus *RpS3*), *RpS3*+puc FDR (p-value for *RpS3*+puc fold change adjusted for multiple testing with the Benjamini-Hochberg procedure) symbol (Flybase gene symbol) and Fullname (Flybase full gene name).

Supplementary Information

Supplementary Methods

RNAseq data generation and analysis

Total RNA from 50 imaginal wing discs was isolated using an RNeasy Mini kit (Qiagen). In all cases at least two biological replicates were analysed. cDNA libraries were prepared from 1 μg of total RNA using TruSeq Illumina kit, according to the manufacturer's protocol. The libraries were sequenced using Illumina HiSeq2000 or Illumina HiSeq2500 machines, obtaining at least 18 million single-end 50 bp reads per library. The sequencing reads were subsequently mapped to the Flybase transcriptome (version 5.57) using the Burrows-Wheeler Aligner (BWA)[1]. Matching reads were counted per gene. The obtained gene counts were subjected to differential expression analysis using the DESeq package[2] (default settings) with an FDR threshold of 0.1 (Benjamini-Hochberg procedure). Gene Ontology was performed using DAVID Bioinformatics resources[3].

Quantitative PCR

RNA was isolated from 15 wing discs per each sample (four biological replicates per genotype), dissected from female wandering L3 larvae of the indicated genotype. After the dissection wing discs were stored in RNAlater at -20°C (Qiagen Cat. no. 76104). The RNA was isolated using the RNeasy Kit (Qiagen Cat. no. 74104), cDNA was generated using Superscript III (Thermo Fischer Cat. no. 18080093) reverse transcriptase with random hexamer primers and 200ng of

total RNA as the template. qPCR assays were performed on CFX Connect Bio-Rad machine using the iTaq™ Universal SYBR® Green Supermix (Bio-Rad Cat. no. 1725121). The following primer sequences were used: Act5c: aagtaccccattgagcacga, acatacatggcgggtgtgtt; Aldh: gcctggctgatctcatggaac, tgtaggacatgctgtagggct; Cyp6d4: ttgaaaaacactccgggattcc, gcgctctttactcttcacataca; Cyp9f2: ggatggccagaaagtcgagg, acgatggaagccgcatgtag; Gclc: gtgacatattgaaatgggg, ttccttctcattgagctgtgc; MRP: aatcgaaagtatggcgtgcag, ggggaatcgacagcacagt; ref(2)p: aatcgagctgtatcttttccagg, aacgtgcatattgctctcgca.

Supplementary Tables

Supplementary Table 1: *Drosophila* genotypes used in the study.

Fig.	Panel	Genotype	Heat-shock, clone age
1		hs-FLP, stauGFP/+; FRT42D, *mahj*/FRT42D, *mahj*	n/a
		FRT82B, *RpS3*[Plac92], ubi-GFP/+	n/a
		FRT82B, *RpS3**/+	n/a
		FRT42D, $M(2)53^{1}$/+	n/a
2	b	TRE16-dsRED/+	n/a
	c	TRE16-dsRED/FRT42D, $M(2)53^{1}$	n/a
	d	TRE16-dsRED/+; FRT82B, *RpS3*[Plac92], ubi-GFP/+	n/a
	f	hs-FLP; Upd3-Gal4, UAS-GFP/+; FRT82B, *RpS3*[Plac92], ubi-GFP / FRT82B	15 min, 72 h
	g	hs-FLP; Upd3-Gal4, UAS-GFP/UAS-puc; FRT82B, *RpS3*[Plac92], ubi-GFP / FRT82B	15 min, 72 h
	h-i	hs-FLP; tub>CD2>Gal4, UAS-CD8-GFP/ UAS-puc-RNAi; tub-$Gal80^{TS}$/+	30 min, 72 h
	k	hs-FLP; Act>*y*>Gal4, UAS-GFP/+; FRT82B, *RpS3*[Plac92], ubi-GFP / +	10 min, 72 h
	l	hs-FLP; Act>*y*>Gal4, UAS-GFP/UAS-puc; FRT82B, *RpS3*[Plac92], ubi-GFP/ +	10 min, 72 h
	m	hs-FLP; Act>*y*>Gal4, UAS-GFP/UAS-dIAP1; FRT82B, *RpS3*[Plac92], ubi-GFP/ +	10 min, 72 h
	o	hs-FLP; Act>*y*>Gal4, UAS-GFP/+; FRT82B, *RpS3*[Plac92], ubi-GFP / +	10 min, 72 h
3	a-b	esg-Gal4, UAS-GFP/UAS-Puc; FRT82B, RpS3[Plac92], ubiGFP/+	n/a
	c-d	en-Flp; act>STOP>Gal4, UAS-GFP/UAS-puc; FRT82B, RpS3[Plac92], ubiGFP /MKRS	n/a
4	b-c	hs-FLP; 10xSTAT-GFP/+; FRT82B, *RpS3*[Plac92], tub-dsRED / FRT82B	20 min, 48 h
	d-e	hs-FLP; Upd3-Gal4, UAS-GFP/+; FRT82B, *RpS3*[Plac92], tub-dsRED / FRT82B	20 min, 48 h
	f-g	hs-FLP; tub>CD2>Gal4, UAS-CD8GFP/UAS-puc; upd3-lacZ/FRT82B, *RpS3**	12 min, 72 h
	h	UAS-$Dome^{\Delta Cyt}$/en-Gal4, UAS-GFP; +/+	n/a
	i	UAS-$Dome^{\Delta Cyt}$/en-Gal4, UAS-GFP; FRT82B, *RpS3*[Plac92], ubi-GFP/ +	n/a
	k	+/hs-FLP;; FRT82B, *RpS3*[Plac92], tub-dsRED / FRT82B	5 min, 54 h
	l	$Dome^{g0218}$/hs-FLP;; FRT82B, *RpS3*[Plac92], tub-dsRED / FRT82B	5 min, 54 h
	n	$Dome^{g0218}$/hs-FLP;; FRT82B, *RpS3*[Plac92], tub-dsRED / FRT82B<br>+/hs-FLP;; FRT82B, *RpS3*[Plac92], tub-dsRED / FRT82B<br>$Dome^{g0218}$/+;; +/FRT82B<br>+/hs-FLP;; +/ FRT82B	
5	b	hs-FLP; tub>Gal4, UAS-CD8-GFP/+; tub-$Gal80^{TS}$/UAS-Nrf2	n/a
	c-d	hs-FLP; GstD1-GFP/+; FRT82B, *RpS3*[Plac92], tub-dsRED / FRT82B	17 min, 48h
	e-f	en-Gal4, UAS-RFP/GstD1-GFP; UAS-mahjRNAi/+	n/a
	g-h	en-Gal4, UAS-FLP/+; FRT82B, *RpS3*[Plac92], tub-dsRED / FRT82B	n/a
	i-j	en-Gal4, UAS-RFP/+; tub-$Gal80^{TS}$/UAS-mahj-RNAi	n/a
6	a	hh-Gal4/UAS-Nrf2 RNAi	n/a
	b	FRT82B, *RpS3*[Plac92], hh-Gal4/UAS-Nrf2 RNAi	n/a
	c	nub-Gal4/+; +/UAS-Nrf2	n/a
	d	nub-Gal4/+; FRT82B, *RpS3*[Plac92] ubiGFP/UAS-Nrf2	n/a
	e	hs-FLP; tub>Gal4, UAS-CD8-GFP/+; tub-$Gal80^{TS}$/UAS-Nrf2	n/a
	f	hs-FLP; tub>CD2>Gal4, UAS-CD8-GFP/+; tub-$Gal80^{TS}$/+	12 min, 72 h
	g	hs-FLP; tub>CD2>Gal4, UAS-CD8-GFP/+; tub-$Gal80^{TS}$/UAS-Nrf2	12 min, 72 h
	i	hs-FLP; tub>CD2>Gal4, UAS-CD8-GFP/+; tub-$Gal80^{TS}$/UAS-Nrf2	40 min, 72 h
	k	hs-FLP, tub-Gal4, UAS-GFP; tub-$Gal80^{TS}$/+; FRT82B, tub-Gal80 / FRT82B, UAS-Nrf2	12 min, 72 h
	l, n	hs-FLP, tub-Gal4, UAS-GFP; tub-$Gal80^{TS}$/+; FRT82B, *RpS3*[Plac92], tub-Gal80 / FRT82B, UAS-Nrf2	12 min, 72 h
	o-p	hs-FLP; tub>CD2>Gal4, UAS-CD8-GFP/tub-$Gal80^{TS}$; puc[A251]-lacZ/UAS-Nrf2	25 min, 96 h
**Supplementary Figures**			
1	a-b	yw	n/a
	c-d	FRT82B, *RpS3*[Plac92], ubi-GFP/+	n/a
	e-f	FRT42D, $M(2)53^{1}$/+	n/a
	g-h	hs-FLP, stauGFP/+; FRT42D, *mahj*/FRT42D, *mahj*	n/a
	i-j	hs-FLP; FRT82B, *RpS3*[Plac92], ubi-GFP/FRT82B	12 min, 48 h
	k-l	hs-FLP; FRT42D, $M(2)53^{1}$/ FRT42D ubi-GFP	1 h, 72h
2	a	yw	n/a
	b	FRT42D, $M(2)53^{1}$/+	n/a
	c	FRT82B, *RpS3*[Plac92], ubi-GFP/+	n/a
	e	hs-FLP; Upd3-Gal4, UAS-GFP/+; FRT82B, *RpS3*[Plac92], ubi-GFP / FRT82B	15 min, 72 h
	f	hs-FLP; Upd3-Gal4, UAS-GFP/UAS-puc ; FRT82B, *RpS3*[Plac92], ubi-GFP / FRT82B	15 min, 72 h
	h	UAS-dIAP1/+; Hh-Gal4/+	n/a
	i	yw	n/a
	j	yw	n/a
	k	hs-FLP; Act>*y*>Gal4, UAS-GFP/+; FRT82B, *RpS3*[Plac92], ubi-GFP / +	n/a
3	a	hs-FLP; Act>*y*>Gal4, UAS-GFP/+; +/+	10 min, 48 h
	b	hs-FLP; Act>*y*>Gal4, UAS-GFP/+; UAS-puc/+	10 min, 48 h
	d	hs-FLP; tub>CD2>Gal4, UAS-CD8-GFP/+; FRT82B, *RpS3*[Plac92], tub-dsRed/+	12 min, 72 h

	e	hs-FLP; tub>CD2>Gal4, UAS-CD8-GFP/UAS-Bsk^{DN}; FRT82B, *RpS3*[Plac92], tub-dsRed/+	12 min, 72 h
	g	hs-FLP; tub>CD2>Gal4, UAS-CD8-GFP/+; FRT82B, *RpS3*[Plac92], tub-dsRed/+	12 min, 72 h
	h	hs-FLP; tub>CD2>Gal4, UAS-CD8-GFP/+; FRT82B, *RpS3*[Plac92], tub-dsRed/UAS-DrICE RNAi	12 min, 72 h
	j	hs-FLP; tub>CD2>Gal4, UAS-CD8-GFP/+; FRT82B, *RpS3*[Plac92], tub-dsRed/+	12 min, 72 h
	k	hs-FLP; tub>CD2>Gal4, UAS-CD8-GFP/+; UAS-p35/FRT82B, *RpS3*[Plac92], tub-dsRed/+	12 min, 72 h
4	a-b	en-Gal4, UAS-FLP/+; FRT82B, *RpS3*[Plac92], ubi-GFP/ FRT82B	n/a
	c	Upd3-Gal4, UAS-GFP/+	n/a
	d	hs-FLP; Upd3-Gal4, UAS-GFP/+; FRT82B, *RpS3*[Plac92], tub-dsRED / FRT82B	n/a
	e	*upd3*-LacZ/TM6B	n/a
	f	*upd3*-LacZ/FRT82B, *RpS3**	n/a
	g-h	hs-FLP ; tub>CD2>Gal4, UAS-CD8-GFP/CDY; *upd3*-LacZ/FRT82B, *RpS3**	n/a
	i-j	10xSTAT-GFP/UAS-puc; FRT82B, *RpS3*[Plac92], *hh*-Gal4/+	n/a
	k-l	en-Gal4, 10xSTAT-GFP/UAS-Dome$^{\Delta Cyt}$	n/a
5	a	*GstD1*-GFP/+	n/a
	b	*GstD1*-GFP/+; FRT82B, *RpS3*[Plac92], tub-dsRed/+	n/a
	d-e	en-Gal4, UAS-FLP/+; FRT82B, *RpS3*[Plac92], ubi-GFP / FRT82B	n/a
	f-g	en-Gal4, UAS-FLP/+; FRT82B, *RpS3*[Plac92], tub-dsRED / FRT82B	n/a
	h	yw	n/a
6	b	hh-Gal4/UAS-Nrf2	n/a
	c	FRT82B, *RpS3*[Plac92], *hh*-Gal4/UAS-Nrf2	n/a
	d	+/FRT40A ; hh-Gal4/UAS-Nrf2	n/a
	e	UAS-puc/FRT40A ; hh-Gal4/UAS-Nrf2	n/a
	f-g	hs-FLP; tub>CD2>Gal4, UAS-CD8-GFP/+; tub-Gal80^{TS}/UAS-Nrf2	20 min, 72 h
	h-i	hs-FLP; tub>CD2>Gal4, UAS-CD8-GFP/+; tub-Gal80^{TS}/UAS-Nrf2	20 min, 72 h

Supplementary Table 2: GO term analysis for prospective losers signature genes

Biological Process (GO Term)	Count	Fold Enrichment	p-value	p adj.
oxidation reduction	43	2.35	2.00E-07	2.37E-04
response to DNA damage stimulus	13	3.83	1.31E-04	7.49E-02
DNA repair	12	3.99	1.87E-04	7.11E-02
cellular response to stress	16	3.08	1.97E-04	5.67E-02
telomere maintenance	5	11.31	7.65E-04	1.66E-01
telomere organization	5	11.31	7.65E-04	1.66E-01
cellular amino acid biosynthetic process	6	7.02	1.39E-03	2.41E-01
double-strand break repair	5	8.08	2.92E-03	3.91E-01
positive regulation of signal transduction	7	4.66	3.58E-03	4.12E-01
positive regulation of cell communication	7	4.48	4.35E-03	4.37E-01

Legend: Count (number of genes related to the biological term), Fold Enrichment (of genes related to biological process), p-value and adjusted p-value (adjusted using the Benjamini correction).

Supplementary Table 3: Toll pathway genes differentially expressed in loser cells.

Gene symbol	FC mahj	FC RpS3	FC RpS3star
pip	4.4026E-01	4.1607E-01	3.1879E-01
Dif	4.5863E+00	3.6529E+00	6.1728E+00
Traf4	2.4494E+00	1.7019E+00	2.2888E+00
PGRP-SA	4.4884E+00	2.9053E+00	3.6107E+00
spz3	1.5304E+00	1.1501E+00	1.5719E+00
SPE	2.9996E-01	8.7552E-01	4.2304E-01

Legend: FC mahj (fold change in expression levels between *mahj*$^{-/-}$ and WT tissues), FC RpS3 (fold change in expression levels between *RpS3*$^{+/-}$ and WT tissues), FC RpS3* (fold change in expression levels between *RpS3**$^{+/-}$ and WT tissues).

Supplementary Figures

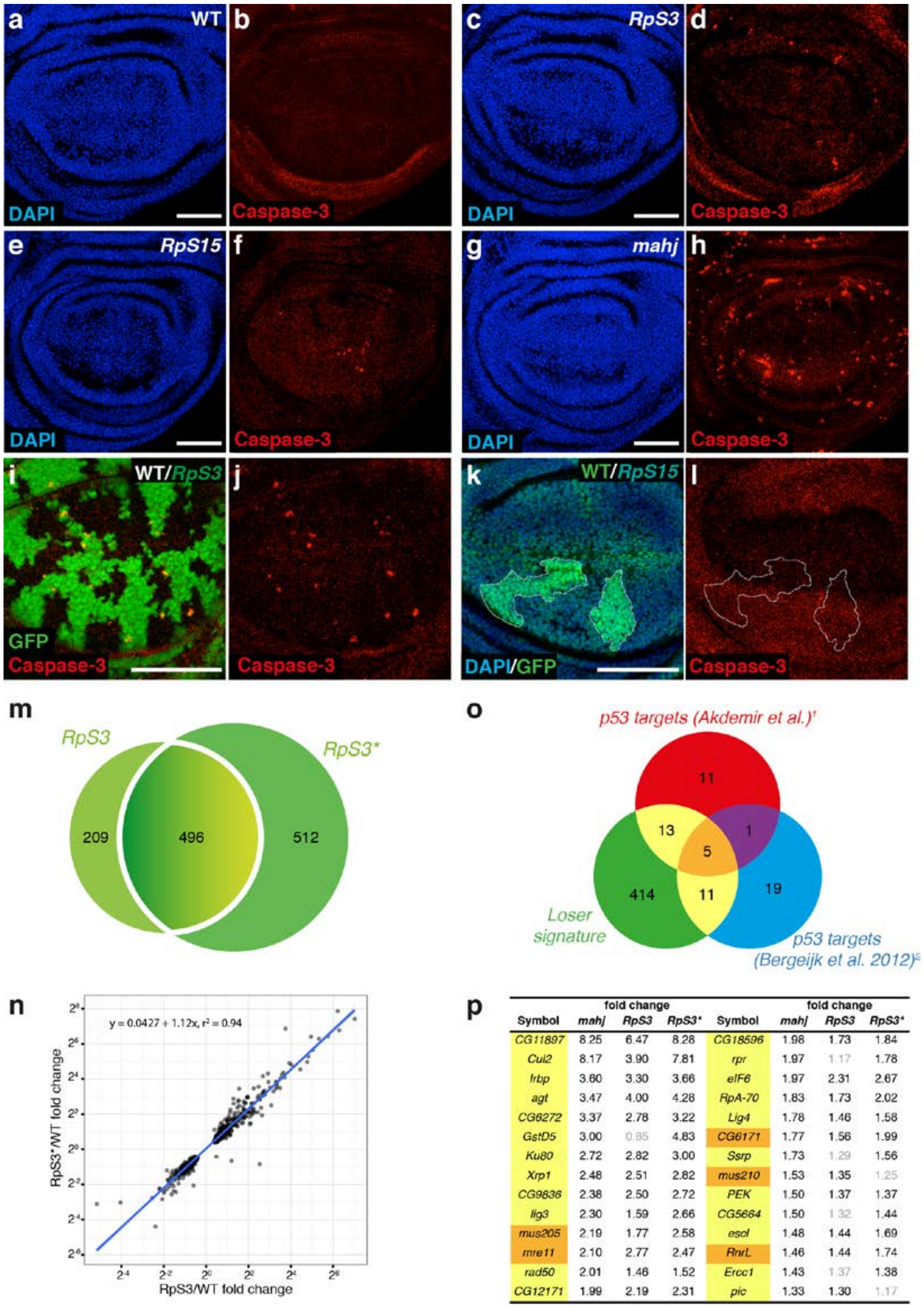

Symbol	fold change *mahj*	fold change *RpS3*	fold change *RpS3**	Symbol	fold change *mahj*	fold change *RpS3*	fold change *RpS3**
CG11897	8.25	6.47	8.28	*CG18596*	1.98	1.73	1.84
Cul2	8.17	3.90	7.81	*rpr*	1.97	1.17	1.78
Irbp	3.60	3.30	3.66	*eIF6*	1.97	2.31	2.67
agt	3.47	4.00	4.28	*RpA-70*	1.83	1.73	2.02
CG6272	3.37	2.78	3.22	*Lig4*	1.78	1.46	1.58
GstD5	3.00	0.85	4.83	*CG6171*	1.77	1.56	1.99
Ku80	2.72	2.82	3.00	*Ssrp*	1.73	1.29	1.56
Xrp1	2.48	2.51	2.82	*mus210*	1.53	1.35	1.25
CG9836	2.38	2.50	2.72	*PEK*	1.50	1.37	1.37
lig3	2.30	1.59	2.66	*CG5664*	1.50	1.32	1.44
mus205	2.19	1.77	2.58	*escl*	1.48	1.44	1.69
mre11	2.10	2.77	2.47	*RnrL*	1.46	1.44	1.74
rad50	2.01	1.46	1.52	*Ercc1*	1.43	1.37	1.38
CG12171	1.99	2.19	2.31	*pic*	1.33	1.30	1.17

Supplementary Figure 1. Analysis of the prospective loser cell molecular signature. (a-h) Representative wing discs, stained with DAPI (blue, a,c,e,g) and anti-cleaved caspase-3 (b,d,f,h) from WT (a-b), *RpS3*$^{+/-}$ (c-d), *RpS15*$^{+/-}$ (e-f), or

mahj$^{-/-}$ (g-h) wing discs dissected from wandering third instar larvae. (i-j) *RpS3*$^{+/-}$ wing discs harbouring *hs*-FLP-induced WT clones (GFP-negative (i), immunostained with anti-cleaved Caspase-3 (red, j)). (k-l) Mosaic wing disc harbouring 1X-GFP *RpS15*$^{+/-}$ cells and 2X-GFP WT cells immunostained for anti-cleaved Caspase-3 (red, l). Note the absence of localised cell death (red). (m) Venn diagram showing the overlap of genes differentially expressed in *RpS3* and *RpS3** compared to wild-type. (n) Linear regression of fold changes of differentially expressed genes in WT vs. *RpS3* and WT vs. *RpS3**; shaded area indicates 95% confidence interval for the fit. (o) Venn diagram showing overlap between loser signature genes and previously identified p53 target genes from two separate studies[4,5]. (p) List of p53 targets found in the loser signature and corresponding fold change increase in each loser genotype. Detailed genotypes for each figure panel are listed in Supplementary Table 1. Scale bars in all supplementary figures represent 50 µm.

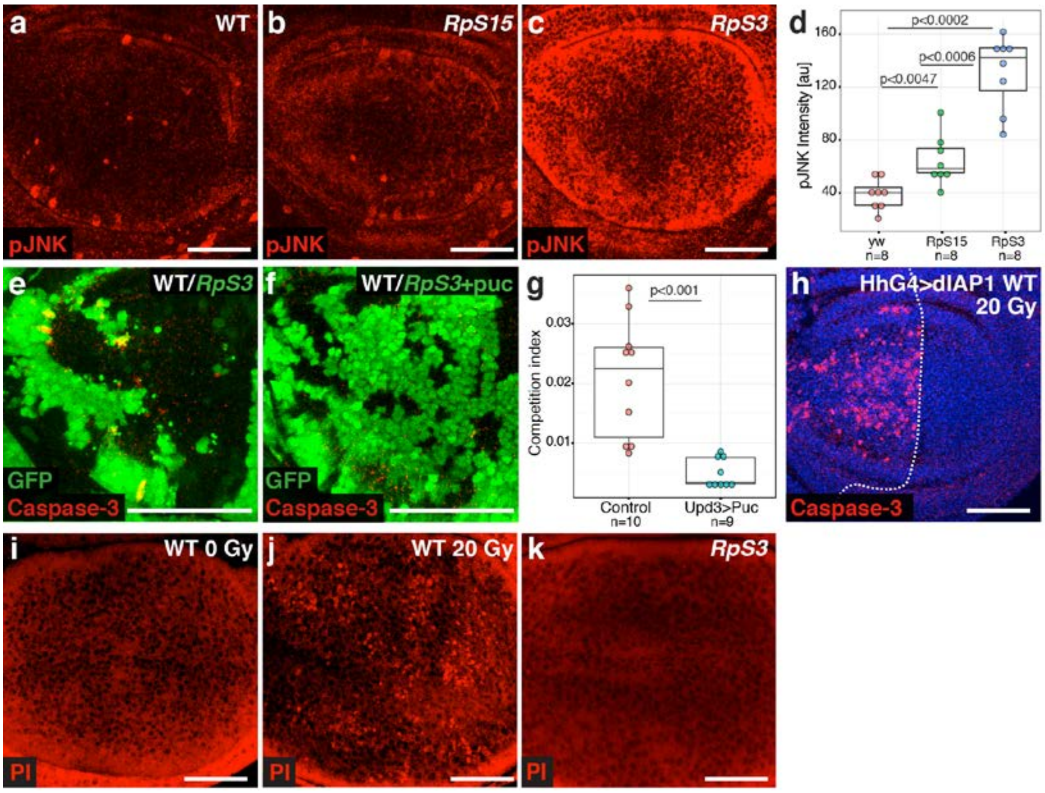

Supplementary Figure 2. Role of JNK signalling in prospective loser cells (a-c) Immunostaining for pJNK (red) in WT (a), *RpS15*$^{+/-}$ (b) or *RpS3*$^{+/-}$ wing discs (c). (d) Quantification of fluorescence intensity from images as in (a-c) for the indicated genotypes, each dot represents a single wing disc. p-values according to a Welch t-test. (e-f) Wing discs harbouring 72-hour-old GFP-negative WT clones within GFP-positive *RpS3*$^{+/-}$ tissue with (f) or without (e) overexpression of *puc* only in the *RpS3*$^{+/-}$ cells (achieved using *upd3*-Gal4 as driver), stained with anti-cleaved Caspase-3 (red). (g) Quantification of the number of dying cells occurring within a 2-cell diameter of the clonal boundary, normalised to the clone perimeter (competition index) for wing discs of the same genotype as in (e-f). Each dot represents a wing disc, p-values according to Welch t-test. (h) X-ray irradiated WT wing disc overexpressing dIAP1 in the P compartment (right side of the A-P boundary labelled with white dotted line) stained with anti-cleaved caspase-3 (red). (i-k) Propidium iodide staining (PI, red) of a WT (i), X-ray

irradiated WT (j) or *RpS3*$^{+/-}$ (k) wing disc. Detailed genotypes for each figure panel are listed in Supplementary Table 1.

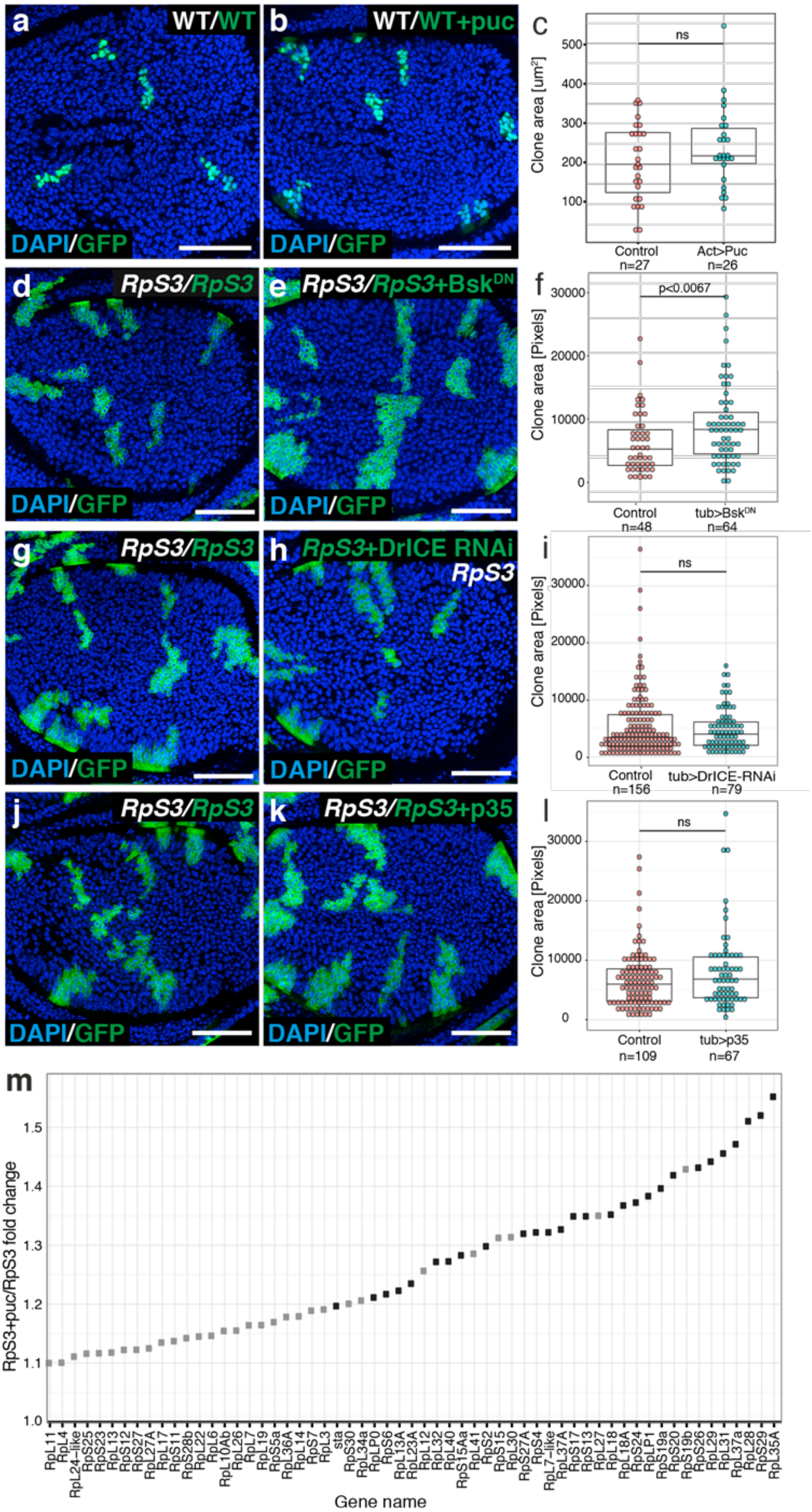

a
WT/WT
DAPI/GFP
b
WT/WT+puc
DAPI/GFP
c
ns
Clone area [um²]
500
400
300
200
100
Control
n=27
Act>Puc
n=26
d
RpS3/RpS3
DAPI/GFP
e
RpS3/RpS3+Bsk^DN
DAPI/GFP
f
p<0.0067
Clone area [Pixels]
30000
20000
10000
0
Control
n=48
tub>Bsk^DN
n=64
g
RpS3/RpS3
DAPI/GFP
h
RpS3+DrICE RNAi
RpS3
DAPI/GFP
i
ns
Clone area [Pixels]
30000
20000
10000
0
Control
n=156
tub>DrICE-RNAi
n=79
j
RpS3/RpS3
DAPI/GFP
k
RpS3/RpS3+p35
DAPI/GFP
l
ns
Clone area [Pixels]
30000
20000
10000
0
Control
n=109
tub>p35
n=67
m
RpS3+puc/RpS3 fold change
1.5
1.4
1.3
1.2
1.1
1.0
RpL11 RpL4 RpL24-like RpS25 RpS23 RpL13 RpS12 RpS27 RpL27A RpL17 RpS11 RpS28b RpL22 RpL6 RpL10Ab RpL26 RpL7 RpL19 RpS5a RpL36A RpL14 RpS7 RpL3 sta RpS30 RpL34a RpLP0 RpS6 RpL13A RpL23A RpL12 RpL32 RpL40 RpS15Aa RpL41 RpS2 RpS15 RpL30 RpS27A RpS4 RpL7-like RpL37A RpS17 RpS13 RpL27 RpL18 RpL18A RpS24 RpLP1 RpS19a RpS20 RpS19b RpS26 RpL29 RpL31 RpL37a RpL28 RpS29 RpL35A
Gene name

Supplementary Figure 3. Effect of JNK inhibition on prospective loser cells

(a-b) WT wing disc harbouring clones overexpressing GFP alone (a) or together with *puc* (b). (c) Size distributions (in μm^2) of clones of the same genotypes as in (a-b), p-values according to a Welch t-test. (d-f) *RpS3*$^{+/-}$ wing disc harbouring clones overexpressing GFP alone (d) or together with a dominant negative form of Basket (Bsk^{DN}, e) and corresponding clone size distributions (f). (g-i) Similar to (d-f), but with clones overexpressing GFP alone (g) or together with knockdown of effector caspase drICE (drICE-RNAi, h). (j-l) Similar to (d-f), but with clones overexpressing GFP alone (j), or together with the caspase inhibitor p35 (k). All p-values calculated by Welch t-test. (m) Dot plot showing the expression of ribosomal genes upregulated greater than 1.1 fold in *RpS3*$^{+/-}$ wing discs upon JNK inhibition, normalized to *RpS3*$^{+/-}$ expression levels (*RpS3*+puc/*RpS3*). Fold changes with FDR>0.1 are in grey. Detailed genotypes for each figure panel are listed in Supplementary Table 1.

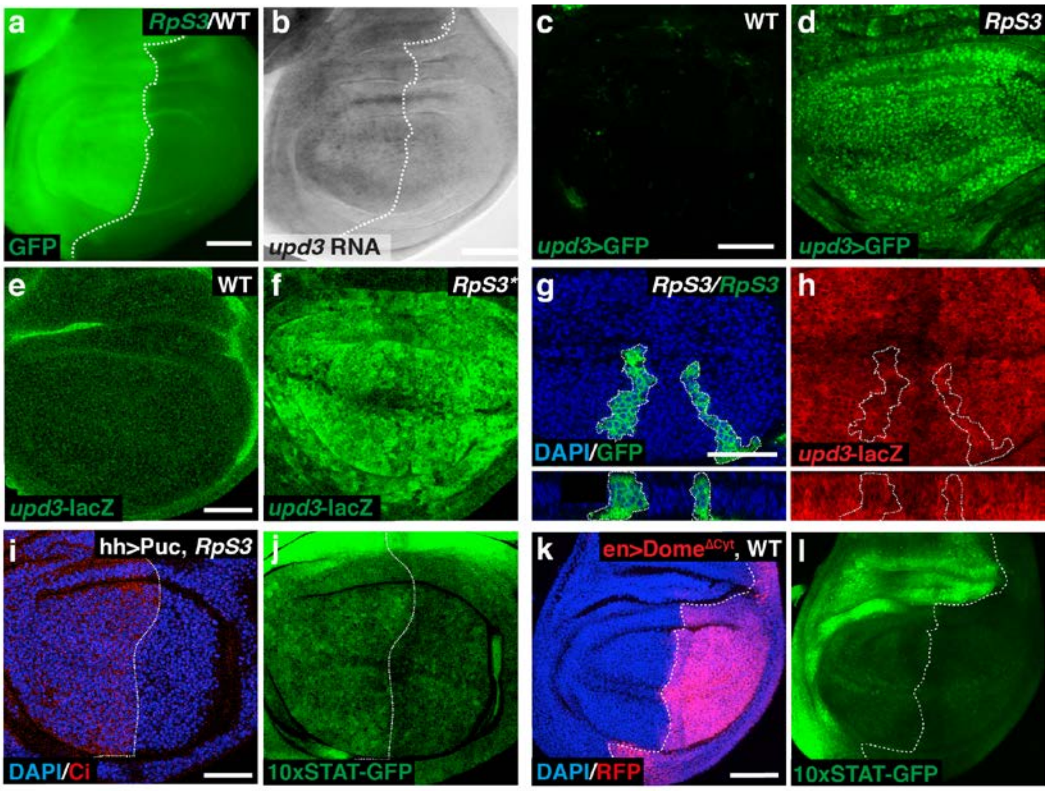

Supplementary Figure 4. Prospective losers upregulate ligands of the JAK/STAT pathway. (a-b) *In situ* hybridisation to detect *upd3* mRNA (black, b) in a wing disc harbouring a GFP-labelled *RpS3*$^{+/-}$ A compartment and a WT P compartment (a) with A-P boundary outlined (white dotted line). (c) *upd3*-Gal4, UAS-GFP reporter activity in a WT wing disc. (d) Upd3-Gal4, UAS-GFP reporter activity in a *RpS3*$^{+/-}$ wing disc. (e) *upd3*-LacZ reporter activity in a WT wing disc. (f) Upd3-lacZ reporter activity in an *RpS3**$^{+/-}$ wing disc. (g-h) *upd3*-LacZ reporter activity (red, h) in *RpS3*$^{+/-}$ wing discs harbouring control GFP-labelled clones (green, g). Both xy (top) and xz projections (bottom) of the same wing disc are presented with clones outlined (white dotted line). (i-j) 10XSTAT-GFP reporter expression (green, j) in a *RpS3*$^{+/-}$ wing disc overexpressing *puc* in the P compartment with the A compartment labelled by immunostaining with anti-Ci (red, i). (k-l) 10XSTAT-GFP reporter expression (green, l) in a WT wing disc expressing RFP (red, k) and a dominant negative JAK/STAT receptor Dome

(Dome$^{\Delta Cyt}$) in the P compartment (A-P boundary labelled with white dotted line).

Detailed genotypes for each figure panel are listed Supplementary Table 1.

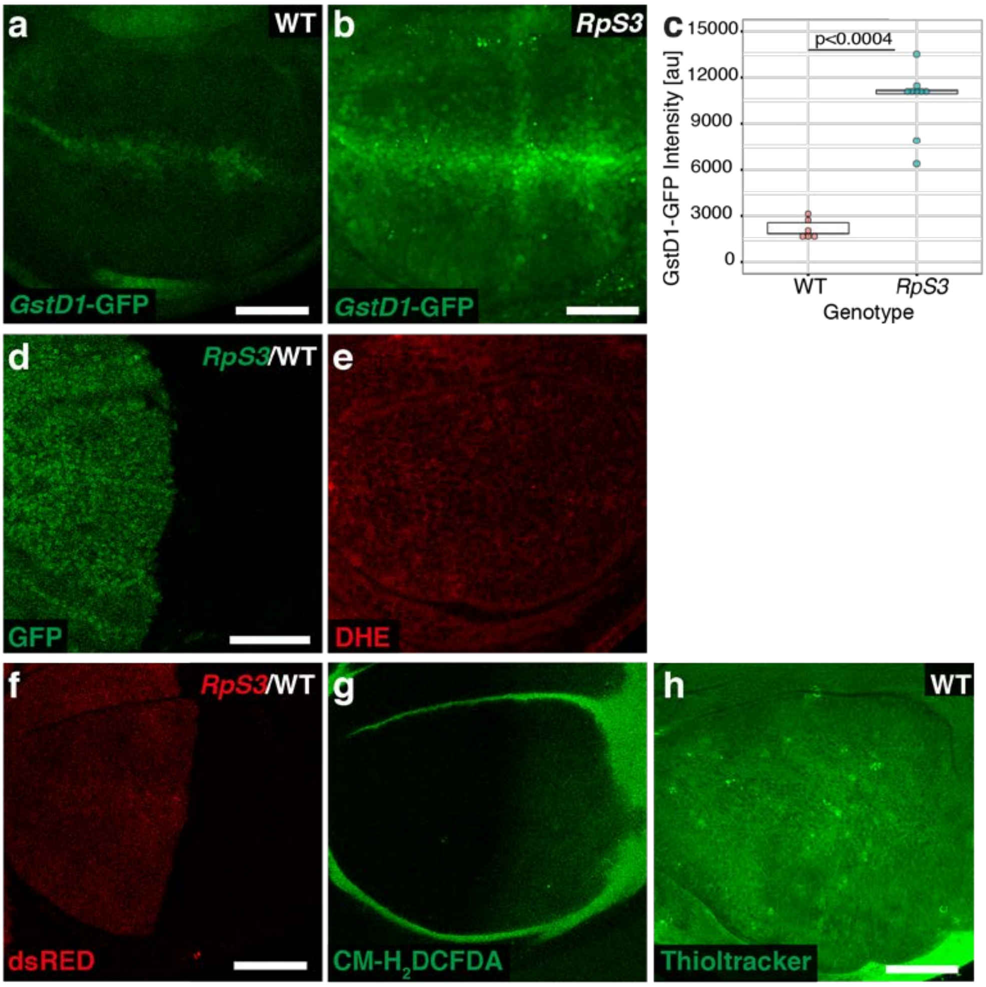

Supplementary Figure 5. Reporters for oxidative stress in *RpS3*$^{+/-}$ cells. (a-b) *GstD1*-GFP reporter activity in WT (a) or *RpS3*$^{+/-}$ wing discs (b). (c) Quantification of fluorescence intensity from images as in (a-b) for the indicated genotypes, each dot represents a single wing disc. p-values according to a Welch t-test. (d-e) DHE staining (red, e) in composite wing disc harbouring a *RpS3*$^{+/-}$ A compartment (GFP-positive, d) and a WT (GFP-negative, d) P compartment. (f-g) CM-H$_2$DCFDA staining (green, g) in composite wing disc harbouring a *RpS3*$^{+/-}$ (dsRED positive, f) A compartment and a WT (dsRED negative, f) P compartment. (h) Thioltracker staining of a WT wing disc. Detailed genotypes for each figure panel are listed in Supplementary Table 1.

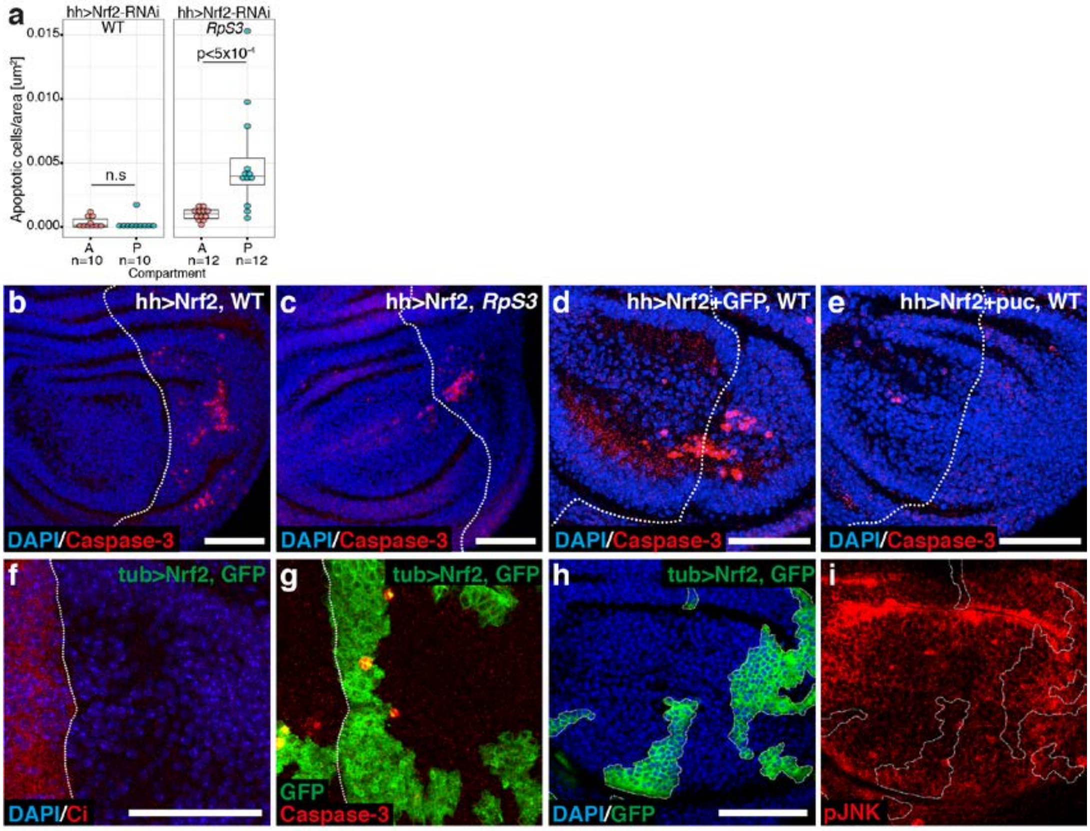

Supplementary Figure 6. Nrf2 overexpression and the loser status. (a) Quantification of the number of apoptotic cells occurring within each compartment of WT (left) or *RpS3*$^{+/-}$ (right) wing discs upon knockdown of Nrf2 (hh>Nrf2-RNAi) in the P compartment. P values calculated by Wilcoxon rank sum test. (b-c) Overexpression of Nrf2 in the P compartment of WT (b) or *RpS3*$^{+/-}$ wing discs (c) stained with anti-cleaved Caspase-3 (red). (d-e) WT wing discs overexpressing Nrf2 and GFP (d) or Nrf2 and *puc* (e) in the P compartment (right side of A-P boundary labelled with white dotted line) stained with anti-cleaved caspase-3 (red). (f-g) WT wing disc harbouring clones overexpressing Nrf2 and GFP (f) and stained with anti-cleaved Caspase-3 (red, g). A-P boundary (labelled with white line) is identified by Ci expression (red, f). Note that cell death does not occur at the A-P boundary. (h-i) Immunostaining for pJNK (red, i)

in WT wing discs harbouring GFP-labelled clones overexpressing Nrf2 (h). Detailed genotypes for each figure panel are listed in Supplementary Table 1.

Supplementary references

1. Li, H. & Durbin, R. Fast and accurate long-read alignment with Burrows-Wheeler transform. *Bioinformatics* **26,** 589–595 (2010).
2. Anders, S. & Huber, W. Differential expression analysis for sequence count data. *Genome Biol.* **11,** R106 (2010).
3. Huang, D. W., Lempicki, R. A. & Sherman, B. T. Systematic and integrative analysis of large gene lists using DAVID bioinformatics resources. *Nat. Protoc.* **4,** 44–57 (2009).
4. Akdemir, F., Christich, A., Sogame, N., Chapo, J. & Abrams, J. M. p53 directs focused genomic responses in Drosophila. *Oncogene* **26,** 5184–93 (2007).
5. van Bergeijk, P., Heimiller, J., Uyetake, L. & Su, T. T. Genome-wide expression analysis identifies a modulator of ionizing radiation-induced p53-independent apoptosis in Drosophila melanogaster. *PLoS One* **7,** e36539 (2012).
